# Small Molecule Inhibitors of KDM5 Histone Demethylases Increase the Radiosensitivity of Breast Cancer Cells Overexpressing JARID1B

**DOI:** 10.3390/molecules24091739

**Published:** 2019-05-04

**Authors:** Simone Pippa, Cecilia Mannironi, Valerio Licursi, Luca Bombardi, Gianni Colotti, Enrico Cundari, Adriano Mollica, Antonio Coluccia, Valentina Naccarato, Giuseppe La Regina, Romano Silvestri, Rodolfo Negri

**Affiliations:** 1 Department of Biology and Biotechnology “C. Darwin”, Sapienza University of Rome, 00185 Rome, Italy; simone.pippa@uniroma1.it (S.P.); valerio.licursi@uniroma1.it (V.L.); luca.bombardi@uniroma1.it (L.B.); 2 Institute of Molecular Biology and Pathology, Italian National Research Council, 00185 Rome, Italy; cecilia.mannironi@uniroma1.it (C.M.); gianni.colotti@uniroma1.it (G.C.); enrico.cundari@uniroma1.it (E.C.); 3 Institute for Systems Analysis and Computer Science “A. Ruberti”, Italian National Research Council, 00185 Rome, Italy; 4 Department of Pharmacy, University “G. d’ Annunzio” of Chieti, Via dei Vestini 31, 66100 Chieti, Italy; adriano.mollica@unich.it; 5 Department of Drug Chemistry and Technologies, Sapienza University of Rome, Laboratory affiliated to Istituto Pasteur Italia Cenci Bolognetti Foundation, Sapienza University of Rome, 00185 Rome, Italy; antonio.coluccia@uniroma1.it (A.C.); valentina.naccarato@uniroma1.it (V.N.); giuseppe.laregina@uniroma1.it (G.L.R.); romano.silvestri@uniroma1.it (R.S.)

**Keywords:** histone demethylase inhibitors, DNA damage, epigenetic drugs, breast cancer

## Abstract

*Background*: KDM5 enzymes are H3K4 specific histone demethylases involved in transcriptional regulation and DNA repair. These proteins are overexpressed in different kinds of cancer, including breast, prostate and bladder carcinomas, with positive effects on cancer proliferation and chemoresistance. For these reasons, these enzymes are potential therapeutic targets. *Methods*: In the present study, we analyzed the effects of three different inhibitors of KDM5 enzymes in MCF-7 breast cancer cells over-expressing one of them, namely KDM5B/JARID1B. In particular we tested H3K4 demethylation (western blot); radio-sensitivity (cytoxicity and clonogenic assays) and damage accumulation (COMET assay and kinetics of H2AX phosphorylation). *Results*: we show that all three compounds with completely different chemical structures can selectively inhibit KDM5 enzymes and are capable of increasing sensitivity of breast cancer cells to ionizing radiation and radiation-induced damage. *Conclusions*: These findings confirm the involvement of H3K4 specific demethylases in the response to DNA damage, show a requirement of the catalytic function and suggest new strategies for the therapeutic use of their inhibitors.

## 1. Introduction

Histone lysine methylation is a post-translational modification that influences many aspects of cell biology, such as transcription, epigenetic inheritance, nuclear architecture and genome stability [1,2]. Unlike histone acetylation, which has a general transcription-promoting action, histone methylation may lead either to transcriptional repression or activation, depending on which residue is involved [3]. Due to the importance of this epigenetic mark, a tight regulation of histone methylation has evolved. The enzymes capable of erasing methyl groups from histones are the histone lysine demethylase (KDMs) and in humans these enzymes are represented by two families of proteins: the lysine-specific histone demethylase (LSD) family and the JmjC domain- containing family also known as the Jumonji histone demethylases (JHDMs) [4,5,6,7]. These two families differ in their catalytic mechanisms. The LSD KDMs are monoamine oxidases whereas JHDMs are hydroxylases that require two cofactors for their function, Fe(II) and 2-oxoglutarate, which are bound in the JMJC catalytic domain. Among the latter ones, particularly interesting are those using H3K4me2 and H3K4me3 as substrates, the KDM5 (or JARID1) enzymes. H3K4 methylation seems to play an important role in development and differentiation and transcriptional regulation [8,9]. Indeed, actively transcribed genes have promoters often marked with H3K4 tri-methylation [10]. Therefore, at first glance, KDM5 enzymes seem to act as transcriptional repressors but, recently, it has been proposed that KDM5 demethylases could also remove tri- and di- methyl groups at enhancer regions. Since H3K4me1 modification combined with acetylated H3K27 (H3K27ac) is predictive of active enhancers, KDM5 enzymes could also have a role in transcriptional activation [11]. The KDM5 subfamily (also known as JARID1 subfamily) consists of four members, KDM5A (JARID1A), KDM5B (JARID1B), KDM5C (JARID1C) and KDM5D (JARID1D) whose deregulation in various kinds of cancer has been widely documented to contribute significantly to tumor initiation and progression [12,13,14,15,16]. Indeed, KDM5B was initially identified as a gene markedly overexpressed in breast cancer even before the discovery of histone lysine demethylases [14]. Nevertheless, it was later observed that the deregulation of this protein is different among the different breast cancer subtypes, thus suggesting a crucial but ambivalent role for KDM5B depending on the cell type context [17]. Later, it was also found to be overexpressed in prostate, lung and bladder carcinoma [13,15]. In the same way, KDM5A was first named RBP2 because it was found to be associated with the master tumor suppressor pRb [18]. By inhibiting pRb activity, KDM5A appears to be a positive regulator of proliferation [19], and it was, in fact, observed to be upregulated in gastric [16] and cervical carcinoma [20]. Recent evidence suggests that KDM5C promotes tumor cells migration and invasion in breast cancer [21]. With regard to the outcome of the action of these proteins on transcription, KDM5B interacts with Cdh4 (a member of NuRD complex) and HDAC1 [22]. The cooperative action resulting by the association of these three catalytic proteins provides a powerful mechanism for a rapid repression of actively transcribed genes involving H3K4 demethylation, lysine deacetylation, and ATPase-mediated chromatin remodeling.

Beside the role in transcriptional regulation, new findings suggest that KDM5 members are involved in the maintenance of genomic stability. KMD5B is enriched at DNA-damage sites after ionizing radiation and its demethylase activity is required for an efficient DNA repair, in contrast with previous observations suggesting a positive role for H3K4 methylation in DNA repair. An interesting model proposed by Li and colleagues [23] tries to establish a connection between the different roles of KDM5B in transcriptional regulation and in DNA repair. During transcriptional activation, PARP1 PARylates KDM5B and prevents it from demethylating H3K4 which would result in shutting off transcription. However, when chromatin is damaged, PARylated KDM5B can be recruited to the damaged sites by histone variant macroH2A1.1 thanks to its PAR binding domain. Local H3K4 demethylation performed by KDM5B is essential for Ku70/80 and BRCA1 recruitment, in the NHEJ and HR pathways, respectively. Moreover, Li and colleagues observed an enhanced and prolonged phosphorylation of H2AX and p53 when cells lacking KDM5B are irradiated. The phosphorylation of these two key players in DNA damage repair (DDR) occurs also in non-irradiated cells, suggesting a crucial role for KDM5B in genome’s integrity, regardless of irradiation. A similar mechanism has been recently proposed for KDM5A as well, even though it is still not clear whether the catalytic activity of this enzyme is necessary for DNA repair or in this case the stimulation of repair is achieved by an indirect mechanism [24].

For the pivotal functions of KDM5 enzymes in different cellular processes, it is important to understand the mechanisms underlying their regulation. Moreover, given the prominent role they have in oncogenesis, they are also promising candidate as therapeutic targets.

In the last decade, an increasing number of epigenetic inhibitors has been developed [25]. Among these, compounds targeting histone lysine demethylase are of great interest because several members of this family of enzymes plays a pivotal role in diseases [26]. Thus, histone lysine demethylases are considered putative drug targets also because we have acquired good knowledge of their active site structure [7,27,28,29]. This feature allows us to develop high-affinity inhibitors [30,31,32]. Small molecules that can regulate site-specific methylation can be used both as chemical tools for studying the function of the epigenetic modifiers and as candidate therapeutic agents for diseases caused by aberrant histone methylation [33,34].

Some inhibitors of KDM5 subfamily of JHDMs have been developed [33,34,35,36,37,38]. Since the catalytic mechanism of these proteins relies on α-ketoglutarate, the inhibitors for JHDMs are mostly compounds mimicking this cofactor [39,40,41], with very few exceptions [28]. Our approach in this work was to compare different compounds with different chemical structure and active in different range of concentrations to find common biological effects which can be correlated with their KDM5 inhibitor actions. In this way we could show that inhibiting the catalytic activity of JARID enzymes increase DNA damage accumulation and confer radio-sensitivity to breast cancer cells.

## 2. Results

### 2.1. RS3195 Is a KDM5 Enzymes Inhibitor That Induces a Strong G2/M Arrest in MCF-7 Cells

We previously set up an in vivo screening system to select H3K4-specific histone demethylase inhibitors which allowed us to find new potential inhibitors of KDM5 enzymes, starting from a library of about 6000 compounds predicted to mimic the structure of α-ketoglutarate [36]. One of the molecules identified, called RS3195, is shown in Table 1. 

Further analysis showed that this compound was able to selectively inhibit KDM5B and KDM5D activity in vitro and to decrease H3K4 demethylation in HeLa cells without affecting methylation of other lysins [36]. Since we were particularly interested in the role of KDM5 enzymes in oncogenesis and transcriptional regulation, we decided to focus our analysis on the effects of our compound on MCF-7 breast cancer cell line in which KDM5B over-expression is a crucial feature because it has wide effects on proliferation, transcription regulation and drug resistance [42]. To assess the ability of RS3195 to inhibit KDM5 enzymes, we treated MCF-7 cells with three different concentrations of inhibitor (1 μM, 10 μM and 30 µM). Since in humans the only enzymes accountable for the demethylation of tri-methylated H3K4 are KDM5 histone demethylases, we monitored the levels of H3K4me3 upon RS3195 treatment. As previously observed in HeLa cells [36], RS3195 induces a slight increase in H3K4me3 levels in bulk chromatin of MCF-7 as well (SM, Figures S1 and S2), although, due to high variability, this effect is not statistically significant. However, we noticed that RS3195 strongly affects cell cycle dynamics of MCF-7 at 30 µM, inducing a clear increase of cells in G2/M (SM Figure S3). A similar result was previously observed in HeLa cells [36], suggesting a cytostatic effect of this compound at high concentrations. No cell cycle perturbation was observed at 1 µM, while at 10 µM we observe a slight but significant increase of G0/G1 population which was not previously detected in HeLa cells. As already observed in HeLa cells treated with 30 µM RS3195 there is also a consistent increase of subG1 cells (see SM Figure S9) which could represent a fraction of apoptotic cells.

### 2.2. KDM5 Enzymes Inhibition Does Not Significantly Change the Transcriptome of MCF-7 Cells

To define a transcriptomic signature of H3K4 tri-methylation and dissect the role of KDM5 enzymes in transcriptional regulation and oncogenesis, we performed RNA-seq on MCF-7 cells treated with RS3195. To minimize the cytostatic effect of our inhibitor, we treated cells with a concentration of 10 μM which has a limited effect on cell cycle dynamics as compared with the highest dose. We found some genes significantly modulated upon treatment (SM Table S1) but a complete functional analysis (SM Figures S4 and S5) showed no remarkable alterations in specific gene expression patterns. This limited effect on gene transcription is in accordance with the data provided by Yamamoto and colleagues [42] who observed only modest variations of transcript levels in siKDM5B transfected breast cancer cells, especially in luminal breast cancer cells. Thus, they suggested that KDM5B is not a strong transcriptional repressor but rather a fine-tuning regulator of cell type-specific H3K4 methylation and transcript levels. However, we noticed that the top up-regulated genes (SM Table S1), *CYP1A1*, *CYP1B2*, *ALDH1A3* and *AHRR*) were all known to be involved in the aryl hydrocarbon receptor (AhR) response [43,44,45], a signaling pathway activated by xenobiotics, suggesting that the most significant transcriptomic changes caused by our compound could be due to its potential toxicity. We verified the activation of AhR pathway by analyzing levels of *CYP1A1* and *AHRR* transcripts by Real Time PCR in MCF-7 cells treated with RS3195. This analysis confirmed that RS3195 induces the genes involved in AhR pathway even in a minor extent as compared with the main known elicitor of AhR, TCDD (SM Figure S3). Stimulation of AhR response could be involved in the cell cycle defects induced by RS3195 since the AhRR gene was previously shown to repress the growth of MCF-7 cells affecting transcriptional and/or posttranscriptional regulations of estrogen responsive and cell cycle-related genes [46].

### 2.3. Designing RS 5033, a Selective KDM5 Enzymes Inhibitor with No Effects on Cell Cycle Dynamics

**Figure 1:**
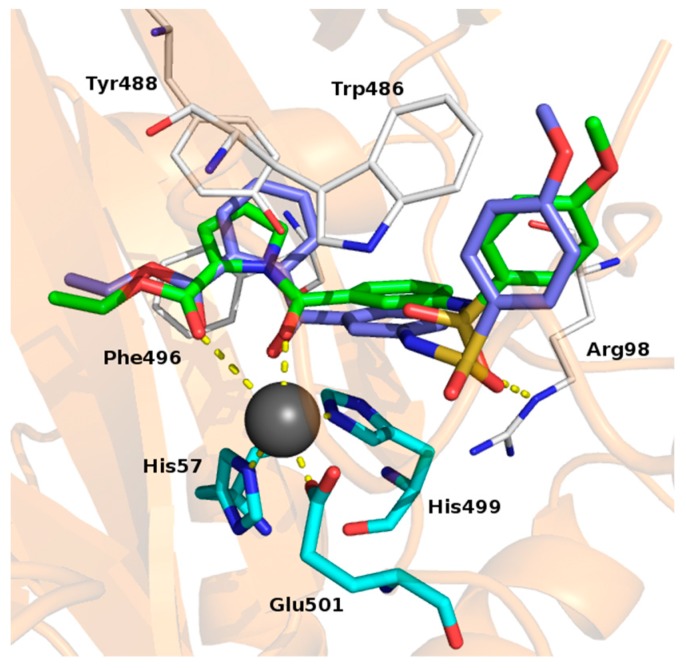
Proposed binding mode for RS3195 (green) and RS5033 (magenta). The iron atom is reported as grey sphere; the coordinating residues are depicted as cyan sticks and the coordination bond as yellow dot lines. The residues involved in interactions with the inhibitors are reported as white lines. H-bond is reported as yellow dot lines.

To gain more insight the RS3195 binding mode we performed docking experiments. The binding mode of the inhibitor was evaluated at the α-KG binding pocket of the KDM5B crystal structure [47,48]. Analyses of the docking poses led us to highlight some key binding interactions: (i) the oxygen atoms of the carboxylate and of the carbonyl al position 1 of the pyrrole formed coordination contacts with the catalytic iron atom; (ii) the pyrrole nucleus formed staking interactions with the aromatic rings of the Tyr488 and Phe496; (iii) the sulfonamide oxygen atom was involved in H-bond with Arg98; (iv) the terminal phenyl ring formed staking contacts with Trp486 (Figure 1).

A test set of RS3195 analogues was synthesized and evaluated by Surface Plasmon Resonance to confirm the proposed binding mode and to draw some structure activity relationships Compounds lacking either the carboxylic moiety (RS3152) or the pyrrole-2-carboxylic group (RS3183) were predicted to be less active than RS3195. Indeed, both compounds do not possess the iron chelating moieties. We aimed to strengthen the aromatic interactions by replacing the pyrrole nucleus with a phenyl ring (RS5033). Finally, we moved the R_2_ substituent (Table 1) from position 3 to position 4 of the central phenyl ring (RS4995). All the new compounds were evaluated by docking experiments. Indeed, derivatives RS4995 and RS5033 shared the same binding mode of RS3195, whereas RS3152 and RS3183 showed distinct binding mode. 

**Figure 2:**
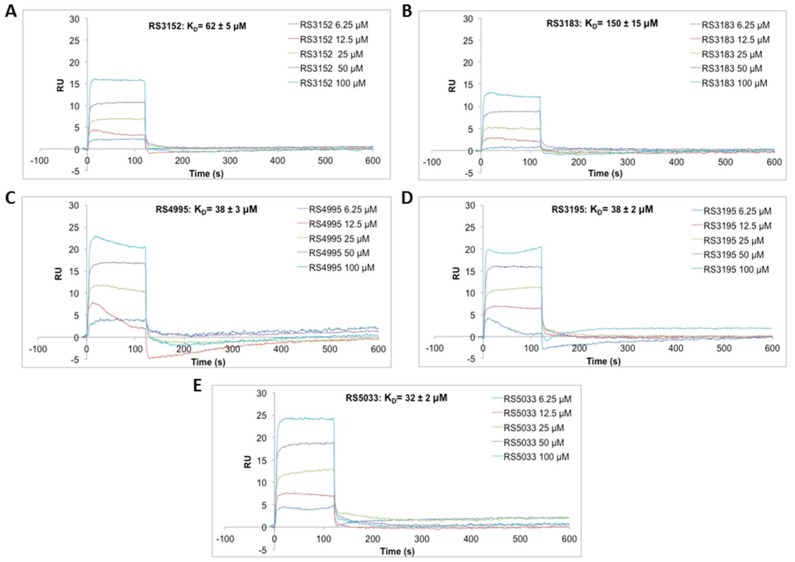
SPR sensorgrams showing the interaction of KDM5D immobilized on a COOH5 sensorchip between inhibitors RS3152 (**A**), RS3183 (**B**), RS4995 (**C**), RS3195 (**D**), RS5033 (**E**) in buffer HSP-1%D) at the following concentrations: 6.25 μM; 12.5 μM; 25 μM; 50 μM; 100 μM. The increase in RU relative to baseline indicates complex formation; the plateau region represents the steady-state phase of the interaction, whereas the decrease in RU after 120 s represents dissociation of analytes from immobilized KDM5D after injection of buffer HSP-1%D.

We tested the relative affinity of these molecules for KDM5D enzyme by Surface Plasmon Resonance (SPR) at various concentrations of compounds (Figure 2; SM Figure S6) and, as predicted, RS3152 and RS3183 showed lower affinity for KDM5 compared to RS3195. RS4995 showed a similar affinity whereas RS5033 proved to be the most affine compound for the catalytic site of the enzyme. The SPR results were in good agreement with the proposed binding modes. The experimental data highlighted the key role of the moieties that bound the catalytic iron atom. 

**Figure 3:**
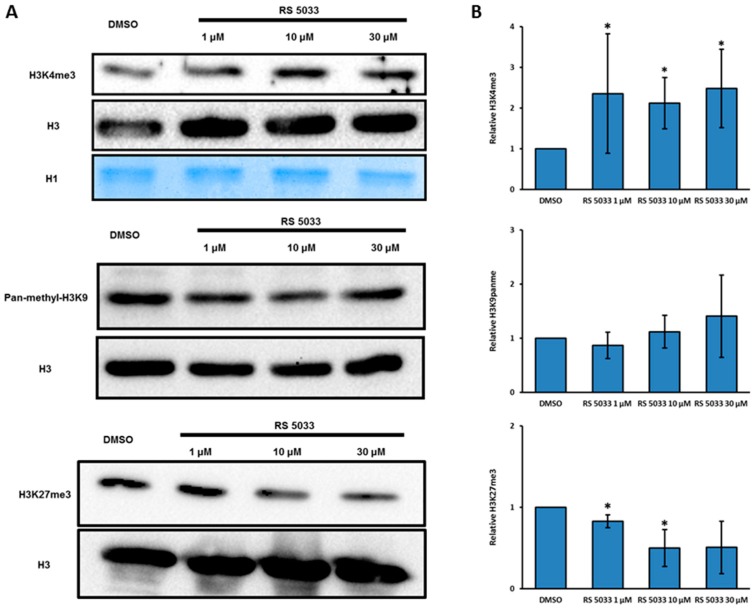
RS 5033 is a selective KDM5 enzymes inhibitor. (**A**) Western blotting analysis of H3K4me3, pan-methyl-H3K9 and H3K27me3 levels in MCF-7 cells after 24 h of incubation with three different concentrations of RS 5033. Shown images are representative from independent experiments. (**B**) Quantification of independent western blotting experiments indicating a strong and significant increase of H3K4me3 levels upon treatment with all three concentrations of RS 5033 (*n* = 6). H3K9me3 levels are not affected (*n* = 5) whereas H3K27me3 levels decrease upon treatment (*n* = 3). Data are normalized to DMSO control and represented the mean ± SD of relative modified histone levels. Statistical significance was assessed according to two-tailed paired Student’s *t* test. * *p* < 0.05.

When tested in vitro (see Materials and Methods) RS5033 proved to be slightly more active than RS3195 (Table 2). Hence, we tested the in vivo effect of different concentration of RS5033 (1 μM, 10 μM and 30 μM) on H3K4 trimethylation and on the cell cycle dynamics in MCF-7 cells. Western blotting analysis on bulk chromatin demonstrated that RS5033 induces a more remarkable and significant increase of H3K4me3 compared to RS3195 (Figure 3A,B). 

Moreover, this compound does not increase H3K9 and H3K27 methylation levels, suggesting a predominant inhibitory action of RS5033 on KDM5 subfamily rather than a general effect on all JHDMs (Figure 3A,B). At higher concentrations, H3K27 tri-methylation seems even to decrease, possibly due to regulatory cross-talk effects. To assess whether RS5033 perturbs cell cycle dynamics as previously observed with RS3195, we performed a flow cytometry analysis on MCF-7 cells treated with 10 μM and 30 μM of RS 5033. The strong increase of cells in G2/M and of the subG1 fraction noted upon treatment with 30 μM of RS3195 were not observed when MCF-7 cells were treated with RS5033 (SM Figures S7–S9). Moreover, Real Time PCR analysis indicated that RS5033 treatment induces only a slight increase the genes involved in AhR pathway that were found to be upregulated upon RS3195 treatment, suggesting that RS5033 stimulates this pathway to a much lesser extent compared to RS3195 (SM Figure S10). Based on these results, RS5033 proved to be a selective inhibitor of KDM5 enzymes that does not affect cell cycle nor significantly stimulates AhR response.

**Figure 4:**
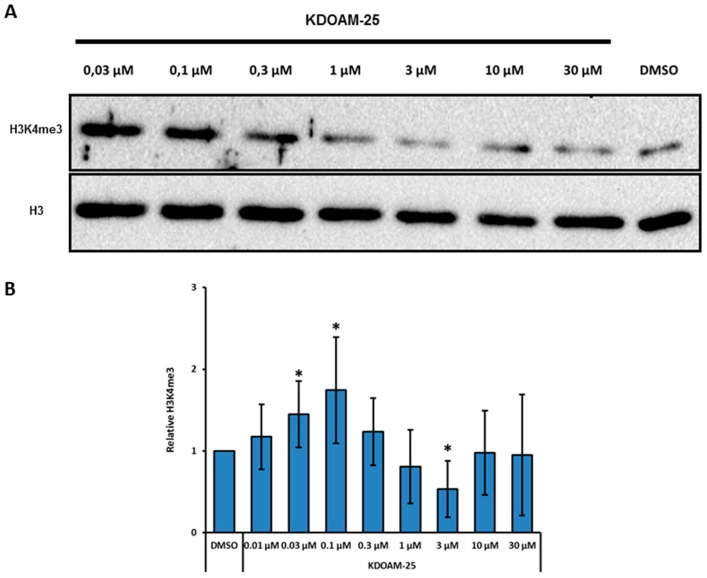
KDOAM-25 is a KDM5 enzymes inhibitor that increases H3K4me3 levels at lower concentration in MCF-7 cells. (**A**) Western blotting analysis of H3K4me3 levels in MCF-7 cells incubated for 24 hours with different concentrations of KDOAM-25. Shown image is representative of four independent experiments. (**B**) Quantification of western blot experiments, showing H3K4m3 levels upon KDOAM-25 treatment. Data are normalized to DMSO control and represented as the mean ± SD of relative H3K4me3 levels (*n* = 4). Statistical significance was assessed according to two-tailed paired Student’s *t* test. * *p* < 0.05.

We also tested KDOAM-25 [49], a recently characterized potent and specific KDM5 enzymes inhibitor with biochemical half maximal inhibitory concentration values of <100 nM for KDM5A-D in vitro. This compound proved to be highly selective toward other 2-OG oxygenases sub-families, and to exert no off-target activity on a panel of 55 receptors and enzymes [49]. KDOAM-25 was reported to inhibit H3K4me3 demethylation at transcription start sites and to block proliferation of MM1S multiple myeloma cells [49]. In our hands the compound leads to a modest (around 1.5-fold) but significant increase of H3K4 tri-methylation in MCF7 bulk chromatin at concentrations 0.03–1 μM, while the effect is lost at higher concentrations (Figure 4A,B). As RS 5033 also this compound does not show any significant effect on cell cycle in the same range of concentrations (not shown).

### 2.4. KDM5 Enzymes Inhibition Increases Breast Cancer Cells Sensitivity to Ionizing Radiation

**Figure 5:**
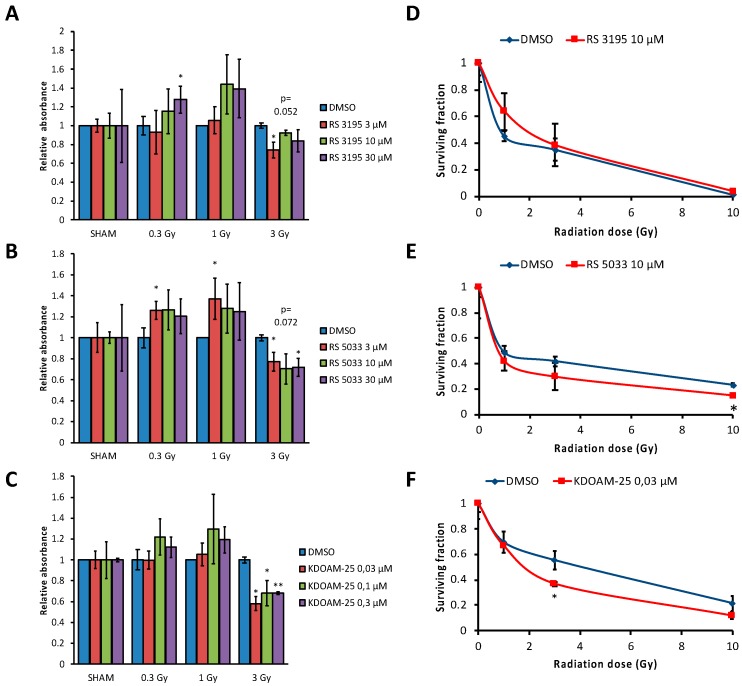
KDM5 enzymes inhibition increases breast cancer cells sensitivity to ionizing radiation. (**A**–**C**) Viability assay on MCF-7 cells irradiated and treated with three different concentration of RS 3195 (**A**), RS5033 (**B**) or KDOAM-25 (**C**). The histogram reports the mean of relative absorbance measured at 450 nm ± SD from three independent experiments. In order to appreciate the effect of the inhibitors on cell viability in each dose of radiation compared to DMSO control, the absorbance of each dose of radiation (sham, 0.3, 1 and 3 Gy) was normalized to the corresponding DMSO control and then, for each concentration of inhibitors, these values were normalized to the corresponding sham control of alive cells and represent the average. (**D**–**F**) Clonogenic assay of MCF-7 cells treated with RS 3195 10 μM (**D**), RS5033 10 μM (**E**) or KDOAM-25 0.03 μM (**F**) and irradiated with 0 Gy, 2 Gy 5 Gy or 10 Gy X-rays. After the irradiation, cells are seeded again and incubated for 1 day with inhibitor and stained with methylene blue after 13 days. For every dose it is indicated the mean value of the cell survival ± SD of 3 independent colony forming assays. Data are normalized to corresponding sham controls. Statistical significance was assessed according to two-tailed paired Student’s *t* test. * *p* < 0.05; ** *p* < 0.001.

Besides the well documented role of KDM5 enzymes in transcriptional regulation, recent findings indicate a novel function for KDM5 enzymes in maintaining genome stability. KDM5A and B were reported to play a crucial role in DDR [23,24,50]. To test the hypothesis that inhibiting KDM5 enzymes could impair the ability of the cells to achieve a correct DDR, we irradiated MCF-7 cells with increasing doses of X-ray (0.3 Gy, 1 Gy and 3 Gy) after treatment with three different concentrations of RS3195 (3 μM, 10 μM or 30 μM); RS 5033 (3 μM, 10 μM or 30 μM) or KDOAM-25 (0.03 μM 0.1 μM and 0.3 μM) in the growth medium. After irradiation cell viability was tested by CCK-8 assay (see Materials and Methods, Figure 5A–C). 

This analysis showed that when MCF-7 cells are irradiated with 3 Gy there is a significant reduction (around 20–35%) of viable cells in presence of all the concentrations of RS5033 and KDOAM-25 compared to DMSO control. RS3195 shows a more limited effect. To test if this increase of radio-sensitivity produces effects in proliferative activity, we performed a clonogenic assay [51]. This assay allows to evaluate the differences in the capacity of cells to produce progeny between control untreated cells and cells treated with a chemical compound and exposed to X-ray. By calculation of the plating efficiency and survival fractions after exposure of cells to radiation both in untreated and treated cells, it is possible to set out a radiation dose-response curve for X-ray irradiated cells. MCF-7 cells were treated either with DMSO, 10 μM RS5033, 10 μM RS3195 or 0.03 μM KDMOA-25 and then irradiated with 0 Gy, 1 Gy, 3 Gy or 10 Gy (Figure 5D–F). A slight but significant decrease of proliferative capacity of MCF-7 cells was observed for 3 Gy irradiation dose after RS5033 and for 3 and 10 Gy after KDMOA-25 treatment compared to DMSO control, while no significant effect was observed for the RS3195 treatment. 

To test if cytoxicity of KDM5 inhibitors were related to overexpression of KDM5B which plays a chemoprotective role in MCF7 cells, we tested the effects of the three drugs on other two breast cancer cell lines: MDA-MB231 which does not overexpress KDM5B and T47D which shows a slightly lower expression compared to MCF-7 (SM Figure S11). As showed in SM Figures S12 and S13, while T47D cells show a clear effects of all three molecules at 1 Gy and 3 Gy, in MDA-MB231 cells the effect is lower and limited to 3 Gy irradiation, suggesting that the abundance of KDM5B is important for the role of H3K4 demethylases in radiation response even if it can be partly surrogated by the other KDM5 enzymes. Overall, these observations show that chemical inhibition of KDM5 demethylases increases radio-sensitivity of breast cancer cells, coherent with the proposed hypothesis that these enzymes are involved in resistance to genotoxic damage [23,24,51].

### 2.5. Effects of KDM5 Inhibitors on DNA Damage Accumulation

**Figure 6:**
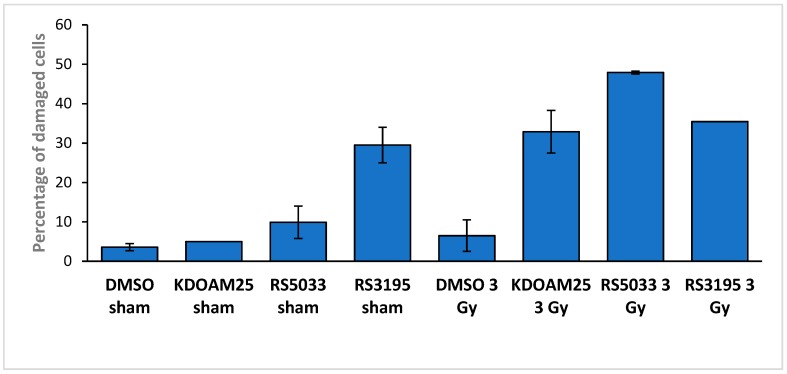
KDM5 enzymes inhibitors increase DNA damage accumulation in irradiated breast cancer cells. The histograms report the % fraction of MCF-7 cells showing a tail long at least as the head diameter in the described conditions. Concentrations were: 3 μM for RS3195 and RS5033 and 0.3 μM for KDOAM-25. DMSO concentration was 0.1% in all samples Data are the average of two independent experiments, variability is indicated (number of observed cells: DMSO sham 54; KDOAM-25 sham 40; RS5033 sham 44; RS3195 sham 98; DMSO 3 Gy 51; KDOAM 3 Gy 68; RS5033 3 Gy 50; RS3195 3 Gy 110).

To test if the cytotoxic effects of KDM5 inhibitors on MCF-7 cells were related to an increase of DNA damage accumulation, we collected cells treated with the three KDM5 inhibitors or DMSO-treated control cells after one hour from irradiation with 3 Gy of ionizing radiation (or sham-irradiation). We then performed COMET assays to assess DNA damage. Stained cells were observed at the fluorescence microscope and we counted the proportion of cells showing evident comet tails (defined as being long at least as the head diameter). Examples of the observed cells are reported in SM Figures S14–S16 and quantitative data are reported in Figure 6. 

Sham irradiated DMSO-treated cells show a very low number of damaged cells. 3 Gy irradiation does not consistently increase the fraction of damaged cells, coherent with a fast and efficient repair of damage in MCF-7 cells. On the other hand, we observe a large fraction (above 30%) of cells with an evident comet tail in cells treated with all three inhibitors and irradiated, confirming that catalytic inhibition of the demethylases can lead to damage accumulation. Interestingly, RS3195 has a strong effect even on sham-irradiated cells, confirming its intrinsic genotoxicity.

### 2.6. Effects of KDM5 Inhibitors on H2AX Phosphorylation

**Figure 7:**
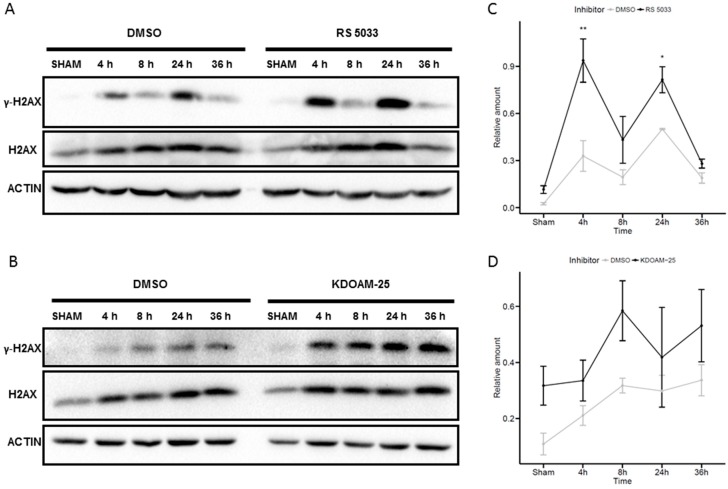
KDM5 inhibition increases H2AX phosphorylation. Western blotting analysis of γ-H2AX levels of irradiated MCF7 cells. MCF-7 cells treated with RS5033 10 μM (**A**) or KDOAM-25 0.03 μM (**B**) are exposed to 0 or 6 Gy of X-rays and irradiated samples are collected after 4, 8, 24 or 36 h. Shown images are representative from at least two independent experiments. (**C**,**D**) Quantification of western blotting experiments, showing an increase in H2AX phosphorylation levels upon RS5033 and KDOAM-25 treatment. Data are represented as the mean ± SD of relative ɤH2AX levels (normalized to total H2AX). Statistical significance was assessed according to one-way ANOVA. * *p* < 0.05; ** *p* < 0.001.

To support the hypothesis that the observed effects of KDM5 inhibitors in enhancing the radio-sensitivity of the MCF-7 cells are due to less efficient DNA damage signaling and repair, we tested the dynamics of H2AX phosphorylation following irradiation in MCF-7 cells treated with the KDM5 inhibitors. We focused on KDOAM-25 and RS5033 whose effects appeared more specifically related to radiation response, while RS3195 which showed less specific toxic effects was left out. As a consequence of genotoxic damage, phosphorylated H2AX (ɤH2AX) accumulates in the nucleus, not exclusively at damaged sites but proportionally to the extent of damage. Its total increase, which can be measured by western blot, is more persistent than its increase at the damaged sites (ɤH2AX foci) and can be used to estimate the total amount of damage [52]. Figure 7A shows that irradiation with 6 Gy of X-rays leads to ɤH2AX accumulation in MCF7 cells. 

Treatment with both RS5033 and KDOAM-25 increases the amount of ɤH2AX both in sham-irradiated and in irradiated cells. When normalized to the amount of H2AX (which is also induced by irradiation) the ɤH2AX increase is statistically significant at 4 h and 24 h in RS5033 treated cells while it is not significant in KDOAM-25 treated cells, due to higher variability (Figure 7B). 

## 3. Discussion

KDM5 enzymes are histone lysine demethylases acting on H3K4. These enzymes, belonging to the family of JHDMs, are hydroxylases capable of erasing H3K4me2 and H3K4me3 marks. For this reason, KDM5 enzymes were initially proposed to be mainly epigenetic regulators of gene transcription and chromatin organization. However recent findings suggest that these enzymes are critical regulators of other important cellular processes, including DNA replication, cell cycle dynamics and DNA damage repair. Importantly, histone lysine demethylases seem even to catalyze other hydroxylation reactions that regulate both protein and nucleic acid-based processes, or to perform novel functions not relying on their demethylase activity, such as acting as molecular scaffold exploiting their chromatin-binding capacity to recruit other proteins and chromatin remodeling activities [53].

In view of all this, developing small molecules capable of inhibiting this subfamily of JHDMs and finding novel mechanisms of regulation of their expression could be helpful to investigate their emerging functions. Since there are growing evidences linking KDM5 enzymes deregulation to several types of tumors, the use of catalytic inhibitors could shed light on the role of these proteins in cancer and pave the way to new strategies of therapy. In particular, KDM5A (JARID1A/RBP2) and KDM5B (JARID1B/PLU1) contribute to cancer cell proliferation, reduce the expression of tumor suppressor genes, promote the development of drug tolerance and maintain tumor initiating cells.

In this work we use a set of different KDM5 catalytical inhibitors to better characterize the role of these enzymes in breast cancer cells, a cellular context in which the relevance of these histone demethylases is well established. First, we found a compound, named RS5033, capable of specifically inhibiting the catalytic mechanism of KDM5 enzymes. This molecule derives from a previous compound (RS3195) identified by a functional screening in *Saccharomyces cerevisiae* [36]. Although RS3195 seemed to consistently increase H3K4me3 levels both in yeast and in HeLa cells, it appears less effective in breast cancer cell line MCF-7 overexpressing KDM5B. Nevertheless, we analyze the effects on MCF-7 transcriptome. The most remarkable modulation caused by RS3195 in the gene expression profile of MCF-7 cells was the up-regulation of the genes involved in aryl-hydrocarbon response, a pathway involved in the regulation of biological responses to planar aromatic (aryl) hydrocarbons, suggesting a potential toxic effect of our compound unrelated to its inhibitory action on H3K4 demethylases. Thus, we moved on RS5033 that proved to be more effective in binding the catalytic site of KDM5 enzymes and not to stimulate the AhR pathway. In a range of concentrations between 1 and 30 µM, RS5033 significantly increases tri-methylation of H3K4 in MCF-7 without increasing methylation of other lysines whose demethylation is not under direct control of KDM5 enzymes. On the contrary, H3K27 tri-methylation seems even to decrease at highest concentration of inhibitor (about two-fold) through a still elusive mechanism. One speculation is the a cross-talk between these two histone modifications exists so that an increase of H3K4me3, which has a positive effect on gene transcription, could indirectly lower the levels of H3K27me3, which is correlated with gene repression. A possible mechanism mediated by a negative impact on PRC2 function has been proposed [54]. We also analyzed the effect on bulk chromatin of KDOAM-25, a new compound that proved to inhibit KDM5 enzymes in vitro with IC_50_ < 100 nM. In MCF-7 cells, this molecule induces a significant increase of H3K4me3 levels at fairly low concentrations (0.03-1 μM) and is therefore at present one of the most promising KDM5 inhibitors. We used RS3195, RS5033 and KDOAM-25 inhibitors to investigate the emerging function of KDM5 enzymes in genome stability maintenance. Thus, we tested their effects on MCF-7 cells radiosensitivity. All three molecules led to a decrease in short-term viability of irradiated MCF-7 cells. As regard long-term effects of radiation, RS5033 and KDOAM-25 treatment decreased the capacity of forming clones in MCF-7 cells exposed to X-ray, whereas such effect appeared not evident in the case of RS3195. It should be noted that in the case of cells treated with RS3195, we consistently obtained a lower number of clones even for sham-irradiated cells (not shown), coherent with its intrinsic toxicity. Next, we used a COMET assay to test damage accumulation on cells irradiated in presence or absence of treatment with inhibitors. The results showed a clear effect of all three KDM5 inhibitors in increasing the number of cells showing damaged DNA at one hour from irradiation compared to solvent treated cells, suggesting that KDM5 demethylase catalytic activity is required for correct damage signaling and repair. Indeed, RS5033 and KDOAM-25 treatment leads to an evident increase of γ-H2AX accumulation in sham-irradiated cells and between 4 and 24 h after irradiation as compared with solvent treated cells. Taken together, this experimental evidence revealed that chemical inhibitors targeting KDM5 enzymes increase sensitivity to spontaneous and X-ray-induced damage. Our results are in line with recent discoveries emphasizing the relevance of histone demethylases for genome stability. 

We recently found two microRNAs which specifically target and repress KDM5B which are strongly down-regulated in breast tumors [55]. These miRNAs can decrease KDM5B protein expression in different breast cancer cell lines, including MCF-7 and at the same time increase their radio-sensitivity [55]. We could not show that the catalytic activity of KDM5B is involved in its radio-protection role, something that we can now conclude. Our work confirmed the involvement of histone demethylases in genome integrity maintenance which could not be limited to JARID enzymes as recently shown [56].

## 4. Materials and Methods

### 4.1. Cell Cultures

MCF-7, T47D and MBA-MB231 human breast cancer cells were purchased from ATCC (ATCC-LGC Standards, ATCC, Manassas, VA, USA) with certification. Cells were cultured for at most 10–12 passages, then replaced by cells from early passages, kept in liquid nitrogen since collection. The presence of Mycoplasma was checked every 6 months using a Mycoplasma PCR detection kit (Sigma-Aldrich, St. Louis, MO, USA). Cells were grown in adherence in high glucose Dulbecco’s Modified Eagle Medium (DMEM) (#FA30WL0101500, Carlo Erba, Cornaredo, Italy) with 1% penicillin/streptomycin (#P11-010, GE Healthcare (PAA), Chicago, IL, USA), 2 mM L-glutamine (#M11-004, PAA, GE Healthcare (PAA), Chicago, IL, USA), 10% previously inactivated fetal bovine serum (#A15-101, GE Healthcare (PAA), Chicago, IL, USA). Cells were maintained at 37 °C and 5% CO_2_ in an incubator (Forma Series II Water Jacketed CO_2_ Incubator Thermo, Waltham, MA, USA) and routinely passaged removing DMEM, washing with PBS (#FA30WL0615500, Carlo Erba) and detaching them using a suitable amount of Trypsin/EDTA (#T4299-100ML, Sigma-Aldrich, St. Louis, MO, USA). KDOAM-25 was purchased from Sigma-Aldrich (#SML1588).

### 4.2. RNA-Sequencing

Total RNA was extracted and quantified as previously described. RNA-Seq libraries preparation and sequencing were performed by the IGA Technology Services (Udine, Italy) using the Illumina TruSeq RNA Sample Preparation Kit V2 (Illumina, San Diego, CA, USA) according to manufacturer’s instructions. The final libraries for single-read sequencing of 50 base pairs were carried out on an Illumina HiSeq2000. Each sample produced about 20 million of reads. Reads quality was evaluated using FastQC (version 0.11.2, Babraham Institute, Cambridge, UK) tool, then reads were mapped to the mouse Ensembl GRCm38 build reference genome using Tophat (version 2.0.12) [57] with the default settings added with the “—no-novel-juncs” option. Gene structure annotations corresponding to the Ensembl annotation release 75 were used to build a transcriptome index and provided to Tophat during the alignment step using the “-G” parameter. The same gene annotation was used to quantify the gene-level read counts using HTSeq-count (version 0.6.1) script, subsequently the differential analyses for gene expression were performed using Bioconductor [58] R (version 3.2.2) (R Core Team, 2015) package DESeq2 (version 1.4.5). The resulting filtered (adjusted *p*-value < 0.1) genes were clustered by enrichment pathway analysis using Bioconductor R packages clusterProfiler [59] and with Gene Ontology Database [60].

### 4.3. Surface Plasmon Resonance (SPR)

Surface Plasmon Resonance (SPR) experiments were carried out using a SensiQ Pioneer system (ICx Nomadics, Stillwater, OK, USA). Immobilization of ligand (KDM5D) was carried out essentially as in Ilari et al. [61]. The sensor chip (COOH5) was activated chemically by a 100 µL injection of a 1:1 mixture of N-ethyl-N′-(3-(diethylaminopropyl)carbodiimide (200 mM) and N-hydroxysuccinimide (50 mM) at a flow rate of 5 µL/min. KDM5D was immobilized on activated sensor chips via amine coupling. The immobilization was carried out in 20 mM sodium acetate at pH 4.5; the remaining unreacted groups were blocked by injecting 1 M ethanolamine hydrochloride (100 µL). The amount of immobilized KDM5D was detected by mass concentration-dependent changes in the refractive index on the sensor chip surface and corresponded to about 11,000 resonance units (RU); an empty flow cell was used as a reference. Analytes, i.e., inhibitors RS3152, RS3183, RS3195, RS4995 and RS5033 were dissolved in DMSO at a concentration of 10 mM and diluted in HSP buffer (10 mM Hepes pH 7.4, 150 mM NaCl + 0.005% surfactant P20) to yield 100 μM inhibitor concentration in HSP + 1% DMSO (HSP-1%D). The analytes were automatically diluted in HSP-1%D and injected on the sensorchip for 120 s at a constant flow (30 µL/min); analyte concentrations were: (1) 6.25 μM; (2) 12.5 μM; (3) 25 μM; (4) 50 μM; (5) 100 μM. The increase in RU relative to baseline indicates complex formation; the plateau region represents the steady-state phase of the interaction, whereas the decrease in RU after 120 s represents dissociation of analytes from immobilized KDM5 after injection of buffer HSP-1%D. The sensorgrams were analysed using the SensiQ Qdat 4.0 program and using simple Scatchard plots.

### 4.4. Chemical Compounds

#### Molecular Modelling

All molecular modelling studies were performed on a MacPro dual 2.66 GHz Xeon running Ubuntu 16LTS. The KDM5B structures were downloaded from PDB [http://www.rcsb.org/.] Pdb code: 5FPL. [47,48]. Hydrogen atoms were added to the protein using Maestro (Small-Molecule Drug Discovery Suite 2015-1, Schrödinger, LLC, New York, NY, USA, 2017) protein preparation wizard [62]. Ligand structures were built with Maestro and minimized using the MMFF94x force field until a rmsd gradient of 0.05 kcal/mol/Å^2^ was reached. The docking simulations were carried out by Plants [63]. The images depicted in the manuscript were generated by Pymol (PyMOL version 1.2r1; DeLanoScientificLLC: SanCarlos, CA http://www.pymol.org/). The residues are numbered according to the crystal structure sequence. 

### 4.5. Enzymatic Inhibition Assay

Inhibition by RS5033 (IC50) was determined by the Epigenase™ JARID Demethylase Activity/Inhibition Assay Kit (Fluorometric) (#P-3083-48 Epigentek, Farmingdale, NY, USA) which is a complete set of optimized reagents, designed for an easy and fast fluorometric measurement of JARID activity or inhibition. The antibody-based, immune-specific method directly detects JARID-converted demethylated products, rather than by- products, in a 96 stripwell microplate format. 

### 4.6. Protein Analysis

#### 4.6.1. Preparation of Whole Cellular Lysate

Preparation of whole cellular lysate was performed with RIPA buffer. DMEM was removed, cells were washed with Dulbecco’s Phosphate Buffered Saline (PBS, #FA30WL0615500, Carlo Erba Reagents) and then resuspended in 80 μL RIPA buffer (#R0278-50ML Sigma-Aldrich) with phosphatase inhibitor (Phosphatase Inhibitor Cocktail 2, #P5726, and Phosphatase Inhibitor Cocktail 3, #P0044, Sigma-Aldrich) and protease inhibitor cocktail (Complete Tablets, Mini EDTA-free, #04693159001, Roche, Mannheim, Germany). Proteins were extracted according to producer’s protocol guidelines and stored at −20 °C until use. BIORAD Quick Start Bradford Dye assay (#5000205) was used to assess protein concentration.

Histone isolation was performed according to acid extraction of histones protocol of Shechter and colleagues [64]. Histone concentration was determined by running 5 μL of samples and known concentrations of commercially available purified calf thymus histones (from Sigma Aldrich Cat. #000000010223565001) on a SDS-PAGE gel. The gel is stained with Coomassie blue G 250 (BIO-RAD Cat. #161-0786) for 1 hour at room temperature and then destained in distilled water. The gel image is acquired with ChemiDoc XRS+ Imaging System (Bio-Rad, Hercules, CA, USA) and the volumes of bands of the samples are compared to standard concentrations of calf thymus histone.

#### 4.6.2. Western Blotting Analysis

Proteins were resolved on a 12% (histones) or 5% (JARID1B) denaturing polyacrylamide gel, using either 4 μg of histone preparation or 40 μg of cell lysate. After electrophoresis, proteins were transferred on a nitrocellulose membrane (Protran™ 0.45 μM NC, # 10600002, GE Healthcare (Amersham™), Chicago, IL, USA) via wet transfer method using a Bio-Rad tank. Transfer efficiency was verified via ponceau staining (#P7767, Sigma-Aldrich). Filters were blocked with either milk (#70166, Sigma-Aldrich) or bovine serum albumin (#A3069, Sigma-Aldrich) and then hybridized over night at 4 °C in gentle rotation with the following antibodies: rabbit anti-H3K4me3 (1:1000, #39915, Active Motif, RRID:AB_2687512); rabbit-anti-H3K27me3 (1:1000, #9733, Cell Signaling Technology, RRID:AB_2616029); rabbit-anti- H3K9pan-methylated (1:1000, #4473, RRID:AB_10544693); rabbit-anti-H3 (1:10,0000, Cat#1791, ABCAM, RRID:AB_302613); rabbit anti-H2AX (1:1000, Cat#7631, Cell Signaling Technology, RRID:AB_10860771), rabbit anti-Phospho-H2AX (1:1000, Cat#9718, Cell Signaling Technology, RRID:AB_2118009), rabbit anti-JARID1B (1:50, Cell Signaling Technology, Cat#3273 RRID: AB_1264191). Goat-anti-Rabbit Horseradish Peroxidase conjugated (1:10,000, #31460, Thermo Fisher Scientific, Waltham, MA, USA RRID:AB_228341) was used as secondary antibody for 1 hour hybridization at room temperature. Blots were scanned and analyzed with a ChemiDoc XRS+ Imaging System (Bio-Rad). The volumes of each band of protein was acquired using Bio-Rad’s ImageLab software by means of volume tools using global quantification. The signal for each protein was normalized to the housekeeping protein and ratios with average control values were determined. For histones as loading control was used either total histone H3 detected by a specific antibody or histone H1 levels by staining the upper part of the gel properly cut before protein transfer on nitrocellulose membrane. 

### 4.7. Gene Expression Analysis

#### 4.7.1. RNA Extraction and Reverse Transcription

Total RNA from treated cells was isolated using miRNeasy^®^ mini kit (#217004, Qiagen, Venlo, Netherlands), according to producer’s protocol guidelines. One μg of total RNA was reverse-transcribed using QuantiTect^®^ Reverse Transcription Kit (#205311, Qiagen), according to producer’s protocol guidelines (Qiagen). 

#### 4.7.2. Quantitative RT-PCR

Relative quantification of gene expression was performed with the 7500 Real Time PCR System (Thermo Fisher Scientific (Applied Biosystems), Waltham, MA, USA) using SensiFAST™ SYBR^®^ Lo-ROX Kit (#BIO 94020, Bioline, London, UK). The relative amount of mRNA expression was determined by means of ΔΔCt method [65], using GAPDH as internal control. Primers were designed using www.ncbi.nlm.nih.gov/tools/primer-blast/ and purchased from Sigma-Aldrich. The following oligos were used:

-3′ CYP1A1 mRNA:
- Fw: 5′- CCCCACAGCACAACAAGAGA-3′
CYP1A1 mRNA:
- Rv: 5′- CAGGGGTGAGAAACCGTTCA-3′
AHRR mRNA:
- Fw: 5′- CAATTACTCAGCAGGAAGGAGC-3′
AHRR mRNA:
- Rv: 5′- CTTGGGGTCAAGGACAAGGTC-3′-3′
GAPDH mRNA:
- Fw: 5′- TCCTCTGACTTCAACAGCGAC-3′
GAPDH mRNA:
- Rv: 5′- CGTTGTCATACCAGGAAAT-3′

### 4.8. Clonogenic Assay

One hour after treatment with inhibitors, cells were exposed to X-ray using a MLG 300/6-D apparatus (Gilardoni, Lecco, Italy) set to 200 V and 6 mA, in order to produce an equivalent absorbed dose of 1cGy/s. Afterwards, cells were harvested, counted and then diluted in inhibitor containing growth medium. Appropriate cell numbers were seeded in quadruplicate according to the doubling time of the cell line and to radiation dose. 24 h after this seeding, the medium with inhibitor was replaced with fresh DMEM and cells were then incubated for 14 days (enough time to allow at least six cell divisions). After this period of growth, cells were washed twice with PBS and then fixed and stained with a suitable volume of a solution made of 0.3% Methylene Blue and 80% Ethanol for 30 min at room temperature. After washing cells twice with ddH_2_O, plates were pictured with ChemiDoc XRS+ Imaging System (Bio-Rad) in colorimetric mode. Colonies were detected using Fiji software [66], with the “Find maxima” function setting a background noise threshold of 3000. Radiation-dose response curveS to X-ray for DMSO and inhibitor samples were calculated using surviving fraction. Plating efficiency and surviving fraction were calculated as follows:

Plating efficiency = number of colonies counted/number of cells plated

Surviving fraction = Plating efficiency/plating efficiency of shame sample

### 4.9. Citotoxicity Assay

The cytoxicity assay was performed with Cell Counting Kit-8 (#96992, Sigma-Aldrich) according to the manufacturer’s instructions. 100 µL of MCF-7 T47D or MDA-MB231 cell suspension (5000 cells/well) was dispensed in a 96-well plate. After 24 h, inhibitor or DMSO was added in growth medium and then cells were irradiated. 48 h post irradiation, 10 μL of CCK-8 solution was added to each well of the plate and incubated again for 1 h. Incubate the plate for 1–4 h in the incubator. The absorbance at 450 nm was read using a VICTOR2 1420 reader (Perkin Elmer Wallac, Waltham, MA, USA). The absorbance at 450 nm of each sample, which is proportional to the number of viable cells, was first normalized on the same value of absorbance of blank (a well with only growth medium). The absorbance of each dose of radiation (sham, 0.3, 1 and 3 Gy) was normalized to the corresponding DMSO control and then, for each concentration of inhibitors, these values were normalized to the corresponding sham control.

### 4.10. Flow-Cytometry

Flow-cytometry analysis of DNA content was carried out using an EPICS xl flow-cytometer (Beckman-Coulter, Brea, CA, USA). DMSO and inhibitor treated MCF-7 cells were trypsinized, pelleted, washed with PBS and finally resuspended in PBS containing 0.1% Triton-X-100 (#A1388.1000, AppliChem, Darmstadt, Germany) and 40 μg/mL propidium iodide (#P-4170, Sigma). After 20 min incubation at 37 °C the samples were analyzed. 10,000 events were acquired for each sample. Acquired data were analyzed using the WinMDI software by Joe Trotter, available at http://facs.scripps.edu.

### 4.11. COMET Assay

The COMET assay [67] was performed using the OxiSelect™ 96-Well Comet Assay Kit (Cell Biolabs, Inc., San Diego, CA, USA) following the manufacturer’s instruction manual (https://www.cellbiolabs.com/sites/default/files/STA-355-comet-assay-kit.pdf).

## Figures and Tables

**Table 1:** Chemical structures for RS compounds 3195, 3152, 3183, 5033 and 4995.

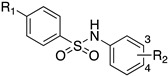			
Compd	R_1_	Position	R_2_
RS3195	CH_3_O	3	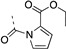
RS3152	CH_3_O	3	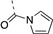
RS3183	CH_3_O	3	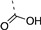
RS5033	CH_3_O	3	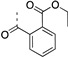
RS4995	Br	4	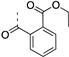

**Table 2:** In vitro IC_50_ of RS 3195, RS 5033 and KDOAM-25 toward KDM5 enzymes.

Compound	IC_50_	Reference
RS 3195	2 μM	Mannironi et al. [36]
RS 5033	1 μM	This work
KDOAM-25	<0.1 μM	Tumber et al. [49]

## Supplementary Materials

The following are available online: Figures S1–S16, Table S1.

## Abbreviations

NuRD	Nucleosome Remodeling and Deacetylase
HDAC	Histone DeACetylase
PARP	Poly (ADP-ribose) polymerase
KDM	histone lysine demethylase
LSD	Lysine-Specific histone Demethylase
JHDMS	Jumonji Histone Demethylases
SPR	Surface Plasmon Resonance
QRT-PCR	Quantitative Reverse Transcription-PCR
IC_50_	half maximal inhibitory concentration

## References

[1] Black J.C., Van Rechem C., Whetstine J.R. (2012). Histone Lysine Methylation Dynamics: Establishment, Regulation, and Biological Impact. Mol. Cell..

[2] Martin C., Zhang Y. (2005). The diverse functions of histone lysine methylation. Nat. Rev. Mol. Cell Biol..

[3] Kouzarides T. (2007). Chromatin Modifications and Their Function. Cell.

[4] Forneris E., Binda C., Vanoni M.A., Mattevi A., Battaglioli E. (2005). Histone demethylation catalysed by LSD1 is a flavin-dependent oxidative process. FEBS Lett..

[5] Karytinos A., Forneris F., Profumo A., Clossani G., Battaglioli E., Binda C., Mattevi A. (2009). A novel mammalian flavin-dependent histone demethylase. J. Biol. Chem..

[6] Klose J.R., Kallin E.M., Khang Y. (2006). JmjC-domain-containing proteins and histone demethylation. Nat. Rev. Gen..

[7] Lohse B., Kristensen J.L., Kristensen L.H., Agger K., Helin K., Gajhede M., Clausen R.P. (2011). Inhibitors of histone demethylases. Bioorg. Med. Chem..

[8] Eissenberg J.C., Shilatifard A. (2010). Histone H3 Lysine 4 (H3K4) Methylation in Development and Differentiation. Dev. Biol..

[9] Lauberth S.M., Nakayama T., Wu X., Ferris A.L., Tang Z., Hughes S.H., Roeder R.G. (2013). H3K4me3 interactions with TAF3 regulate preinitiation complex assembly and selective gene activation. Cell.

[10] Santos-Rosa H., Schneider R., Bannister A.J., Sherriff J., Berstein B.E., Emre N.C., Schreiber S.L., Mellor J., Kouzarides T. (2002). Active genes are tri-methylated at K4 of histone H3. Nature.

[11] Outchkourov N.S., Muiño J.M., Kaufmann K., van Ijcken W.F., Groot Koerkamp M.J., van Leenen D., de Graaf P., Holstege F.C., Grosveld F.G., Timmers H.T. (2013). Balancing of histone H3K4 methylation states by the Kdm5c/SMCX histone demethylase modulates promoter and enhancer function. Cell. Rep..

[12] Blair L.P., Cao J., Zou M.R., Sayegh J., Yan Q. (2011). Epigenetic Regulation by Lysine Demethylase 5 (KDM5) Enzymes in Cancer. Cancers.

[13] Hayami S., Yoshimatsu M., Veerakumarasivam A., Unoki M., Iwai Y., Tsunoda T., Field H.I., Kelly J.D., Neal D.E., Yamaue H. (2010). Overexpression of the JmjC histone demethylase KDM5B in human carcinogenesis: Involvement in the proliferation of cancer cells through the E2F/RB pathway. Mol. Cancer.

[14] Lu P.J., Sundquist K., Baeckstrom D., Poulsom R., Hanby A., Meier-Ewert S., Jones T., Mitchell M., Pitha-Rowe P., Freemont P. (1999). A novel gene (PLU-1) containing highly conserved putative DNA/chromatin binding motifs is specifically up-regulated in breast cancer. J. Biol. Chem..

[15] Xiang Y., Zhu Z., Han G., Ye X., Xu B., Peng Z., Ma Y., Yu Y., Lin H., Chen A.P. (2007). JARID1B is a histone H3 lysine 4 demethylase up-regulated in prostate cancer. Proc. Natl. Acad. Sci. USA.

[16] Zeng J., Ge Z., Wang L., Li Q., Wang N., Bjorkholm M., Jia J., Xu D. (2010). The histone demethylase RBP2 Is overexpressed in gastric cancer and its inhibition triggers senescence of cancer cells. Gastroenterology.

[17] Yamane K., Tateishi K., Klose R.J., Fang J., Fabrizio L.A., Erdjument-Bromage H., Taylor-Papadimitriou J., Tempst P., Zhang Y. (2007). PLU-1 is an H3K4 demethylase involved in transcriptional repression and breast cancer cell proliferation. Mol. Cell..

[18] Defeo-Jones D., Huang P.S., Jones R.E., Haskell K.M., Vuocolo G.A., Hanobik M.G., Huber H.E., Oliff A. (1991). Cloning of cDNAs for cellular proteins that bind to the retinoblastoma gene product. Nature.

[19] Benevolenskaya E.V., Murray H., Branton P., Young R.A., Kaelin W.G. (2005). Binding of pRB to the PHD protein RBP2 promotes cellular differentiation. Mol. Cell..

[20] Hidalgo A., Baudis M., Petersen I., Arreola H., Piña P., Vázquez-Ortiz G., Hernández D., González J., Lazos M., López R. (2005). Microarray comparative genomic hybridization detection of chromosomal imbalances in uterine cervix carcinoma. BMC Cancer.

[21] Wang Q., Wei J., Su P., Gao P. (2015). Histone demethylase JARID1C promotes breast cancer metastasis cells via down regulating BRMS1 expression. Biochem. Biophys. Res. Commun..

[22] Klein B.J., Piao L., Xi Y., Rincon-Arano H., Rothbart S.B., Peng D., Wen H., Larson C., Zhang X., Zheng X. (2014). The histone-H3K4-specific demethylase KDM5B binds to its substrate and product through distinct PHD fingers. Cell Rep..

[23] Li X., Liu L., Yang S., Song N., Zhou X., Gao J., Yu N., Shan L., Wang Q., Liang J. (2014). Histone demethylase KDM5B is a key regulator of genome stability. Proc. Natl. Acad. Sci. USA.

[24] Gong F., Clouaire T., Aguirrebengoa M., Legube G., Miller K.M. (2017). Histone demethylase KDM5A regulates the ZMYND8-NuRD chromatin remodeler to promote DNA repair. J. Cell. Biol..

[25] Heerboth S., Lapinska K., Snyder N., Leary M., Rollinson S., Sarkar S. (2014). Use of Epigenetic Drugs in Disease: An Overview. Genet. Epigenet..

[26] Højfeldt J.W., Agger K., Helin K. (2014). Histone lysine demethylases as targets for anticancer therapy. Nat. Rev. Drug Discov..

[27] McGrath J., Trojer P. (2015). Targeting histone lysine methylation in cancer. Pharmacol. Ther..

[28] Wang L., Chang J., Varghese D., Dellinger M., Kumar S., Best A.M., Ruiz J., Bruick R., Peña-Llopis S., Xu J. (2013). A small molecule modulates Jumonji histone demethylase activity and selectively inhibits cancer growth. Nat. Commun..

[29] Spannhoff A., Hauser A.T., Heinke R., Sippl W., Jung M. (2009). The emerging therapeutic potential of histone methyltransferase and demethylase inhibitors. Chem. Med. Chem..

[30] Hatch S.B., Yapp C., Montenegro R.C., Savitsky P., Gamble V., Tumber A., Ruda G.F., Bavetsias V., Fedorov O., Atrash B. (2017). Assessing histone demethylase inhibitors in cells: Lessons learned. Epigenetics Chromatin.

[31] Thinnes C.C., England K.S., Kawamura A., Chowdhury R., Schofield C.J., Hopkinson R.J. (2014). Targeting histone lysine demethylases—progress, challenges, and the future. Biochim. Biophys. Acta.

[32] McAllister T.E., England K.S., Hopkinson R.J., Brennan P.E., Kawamura A., Schofield C. (2016). Recent Progress in Histone Demethylase Inhibitors. J. Med. Chem..

[33] Itoh Y., Sawada H., Suzuki M., Tojo T., Sasaki R., Hasegawa M., Mizukami T., Suzuki T. (2015). Identification of Jumonji AT-Rich Interactive Domain 1A Inhibitors and Their Effect on Cancer Cells. ACS Med. Chem. Lett..

[34] Gale M., Sayegh J., Cao J., Norcia M., Gareiss P., Hoyer D., Merkel J.S., Yan Q. (2016). Screen-identified selective inhibitor of lysine demethylase 5A blocks cancer cell growth and drug resistance. Oncotarget.

[35] Gehling V.S., Bellon S.F., Harmange J.C., LeBlanc Y., Poy F., Odate S., Buker S., Lan F., Arora S., Williamson K.E. (2016). Identification of potent, selective KDM5 inhibitors. Bioorg. Med. Chem. Lett..

[36] Mannironi C., Proietto M., Bufalieri F., Cundari E., Alagia A., Danovska S., Rinaldi T., Famiglini V., Coluccia A., La Regina G. (2014). An high-throughput in vivo screening system to select H3K4-specific histone demethylase inhibitors. PLoS ONE.

[37] Kristensen L.H., Nielsen A.L., Helgstrand C., Lees M., Cloos P., Kastrup J.S., Helin K., Olsen L., Gajhede M. (2012). Studies of H3K4me3 demethylation by KDM5B/Jarid1B/PLU1 reveals strong substrate recognition in vitro and identifies 2,4-pyridine-dicarboxylic acid as an in vitro and in cell inhibitor. FEBS J..

[38] Sayegh J., Cao J., Zou M.R., Morales A., Blair L.P., Norcia M., Hoyer D., Tackett A.J., Merkel J.S., Yan Q. (2013). Identification of small molecule inhibitors of Jumonji AT-rich interactive domain 1B (JARID1B) histone demethylase by a sensitive high throughput screen. J. Biol. Chem..

[39] Rose N.R., Ng S., Mecinović J., Liénard B.M., Bello S.H., Sun Z., McDonough M.A., Oppermann U., Schofield C.J. (2008). Inhibitor scaffolds for 2-oxoglutarate-dependent histone lysine demethylases. J. Med. Chem..

[40] Luo X., Liu Y., Kubicek S., Myllyharju J., Tumber A., Ng S., Che K.H., Podoll J., Heightman T.D., Oppermann U. (2011). A Selective Inhibitor and Probe of the Cellular Functions of Jumonji C Domain-Containing Histone Demethylases. J. Am. Chem. Soc..

[41] Nie Z., Shi L., Lai C., O’Connell S.M., Xu J., Stansfield R.K., Hosfield D.J., Veal J.M., Stafford J.A. (2018). Structure-based design and discovery of potent and selective KDM5 inhibitors. Bioorg Med. Chem. Lett..

[42] Yamamoto S., Wu Z., Russnes H.G., Takagi S., Peluffo G., Vaske C., Zhao X., Moen Vollan H.K., Maruyama R., Ekram M.B. (2014). JARID1B is a luminal lineage-driving oncogene in breast cancer. Cancer Cell..

[43] Kim S., Dere E., Burgoon L.D., Chang C.C., Zacharewski T.R. (2009). Comparative analysis of AhR-mediated TCDD-elicited gene expression in human liver adult stem cells. Toxicol. Sci..

[44] Lee H.H., Kim W.T., Kim D.H., Park J.W., Kang T.H., Chung J.W., Leem S.H. (2013). Tristetraprolin suppresses AHRR expression through mRNA destabilization. FEBS Lett..

[45] MacPherson L., Ahmed S., Tamblyn L., Krutmann J., Förster I., Weighardt H., Matthews J. (2014). Aryl hydrocarbon receptor repressor and TiPARP (ARTD14) use similar, but also distinct mechanisms to repress aryl hydrocarbon receptor signaling. Int. J. Mol. Sci..

[46] Kanno Y., Takane Y., Izawa T., Nakahama T., Inouye Y. (2006). The inhibitory effect of aryl hydrocarbon receptor repressor (AhRR) on the growth of human breast cancer MCF-7 cells. Biol. Pharm. Bull..

[47] Srikannathasan V., Wohlkonig A., Shillings A., Singh O., Chan P.F., Huang J., Gwynn M.N., Fosberry A.P., Homes P., Hibbs M., Theobald A.J. (2015). Crystallization and initial crystallographic analysis of covalent DNA-cleavage complexes of Staphyloccocus aureus DNA gyrase with QPT-1, moxifloxacin and etoposide. Acta Crystallogr F Struct Biol Commun..

[48] Bavetsias V., Lanigan R.M., Ruda G.F., Atrash B., McLaughlin M.G., Tumber A., Mok N.Y., Le Bihan Y.V., Dempster S., Boxall K.J. (2016). 1.8-Substituted Pyrido[3,4-D]Pyrimidin-4(3H)-One Derivatives as Potent, Cell Permeable, Kdm4 (Jmjd2) and Kdm5 (Jarid1) Histone Lysine Demethylase Inhibitors. J. Med. Chem..

[49] Tumber A., Nuzzi A., Hookway E.S., Hatch S.B., Velupillai S., Johansson C., Kawamura A., Savitsky P., Yapp C., Szykowska A. (2017). Potent and Selective KDM5 Inhibitor Stops Cellular Demethylation of H3K4me3 at Transcription Start Sites and Proliferation of MM1S Myeloma Cells. Cell. Chem. Biol..

[50] Penterling C., Drexler G.A., Böhland C., Stamp R., Wilke C., Braselmann H., Caldwell R.B., Reindl J., Girst S., Greubel C. (2016). Depletion of Histone Demethylase Jarid1A Resulting in Histone Hyperacetylation and Radiation Sensitivity Does Not Affect DNA Double-Strand Break Repair. PLoS ONE.

[51] Rafehi H., Orlowski C., Georgiadis G.T., Ververis K., El-Osta A., Karagiannis T.C. (2011). Clonogenic assay: Adherent cells. J. Vis. Exp..

[52] Ding D., Zhang Y., Wang X., Zhang X., Gao Y., Yin L., Li Q., Li J. (2016). Induction and inhibition of the pan-nuclear gamma-H2AX response in resting human peripheral blood lymphocytes after X-ray irradiation. Cell Death Discovery.

[53] Dimitrova E., Turberfield A.H., Klose R.J. (2015). Histone demethylases in chromatin biology and beyond. EMBO Rep..

[54] O’Meara M.M., Simon J.A. (2012). Inner workings and regulatory inputs that control Polycomb repressive complex 2. Chromosoma.

[55] Mocavini I., Pippa S., Licursi V., Paci P., Trisciuoglio D., Mannironi C., Presutti C., Negri R. (2019). JARID1B expression and its function in DNA damage repair are tightly regulated by miRNAs in breast cancer. Cancer Sci..

[56] Rath B.H., Waung I., Camphausen K., Tofilon P.J. (2018). Inhibition of the histone H3K27 demethylase UTX enhances tumor cell radiosensitivity. Mol. Cancer Ther..

[57] Trapnell C., Roberts A., Goff L., Pertea G., Kim D., Kelley D.R., Pimentel H., Salzberg S.L., Rinn J.L., Pachter L. (2012). Differential gene and transcript expression analysis of RNA-seq experiments with TopHat and Cufflinks. Nat. Protoc..

[58] Gentleman R.C., Carey V.J., Bates D.M., Bolstad B., Dettling M., Dudoit S., Ellis B., Gautier L., Ge Y., Gentry J. (2004). Bioconductor: Open software development for computational biology and bioinformatics. Genome Biol..

[59] Yu G., Wang L.G., Han Y., He Q.Y. (2012). clusterProfiler: An R Package for Comparing Biological Themes Among Gene Clusters. OMICS J. Integr. Biol..

[60] Ashburner M., Ball C.A., Blake J.A., Botstein D., Butler H., Cherry J.M., Davis A.P., Dolinski K., Dwight S.S., Eppig J.T. (2000). Gene Ontology: Tool for the unification of biology. Nat. Genet..

[61] Ilari A., Fiorillo A., Poser E., Lalioti V.S., Sundell G.N., Ivarsson Y., Genovese I., Colotti G. (2015). Structural basis of Sorcin-mediated calcium-dependent signal transduction. Sci Rep..

[62] Sastry G.M., Adzhigirey M., Day T., Annabhimoju R., Sherman W. (2013). Protein and ligand preparation: Parameters, protocols, and influence on virtual screening enrichments. J. Comput Aided Mol. Des..

[63] Korb O., Stützle T., Exner T.E. (2009). Empirical scoring functions for advanced protein-ligand docking with PLANTS. J. Chem. Inf. Model..

[64] Shechter D., Dormann H.L., Allis C.D., Hake S.B. (2007). Extraction, purification and analysis of histones. Nat. Protoc..

[65] Livak K.J., Schmittgen T.D. (2001). Analysis of relative gene expression data using real-time quantitative PCR and the 2(-Delta Delta C(T)) Method. Methods.

[66] Schindelin J., Arganda-Carreras I., Frise E., Kaynig V., Longair M., Pietzsch T., Preibisch S., Rueden C., Saalfeld S., Schmid B. (2012). Fiji: An Open-Source Platform for Biological-Image Analysis. Nat. Methods.

[67] Olive P.L., Banath J.P., Durand R.E. (1990). Heterogeneity in radiation induced DNA damage and repair in tumor and normal cells using the “Comet” assay. Radiat. Res..

