# Supplementary material for: Small Molecule Inhibitors of KDM5 Histone Demethylases Increase the Radiosensitivity of Breast Cancer Cells Overexpressing JARID1B
Source: Molecules. 2019 May 4;24(9):1739. doi: 10.3390/molecules24091739 (PMC6540222; doi:10.3390/molecules24091739)
Supplement: Supplementary file 1 [file molecules-24-01739-s001.zip › supplementary.pdf]

SM Description

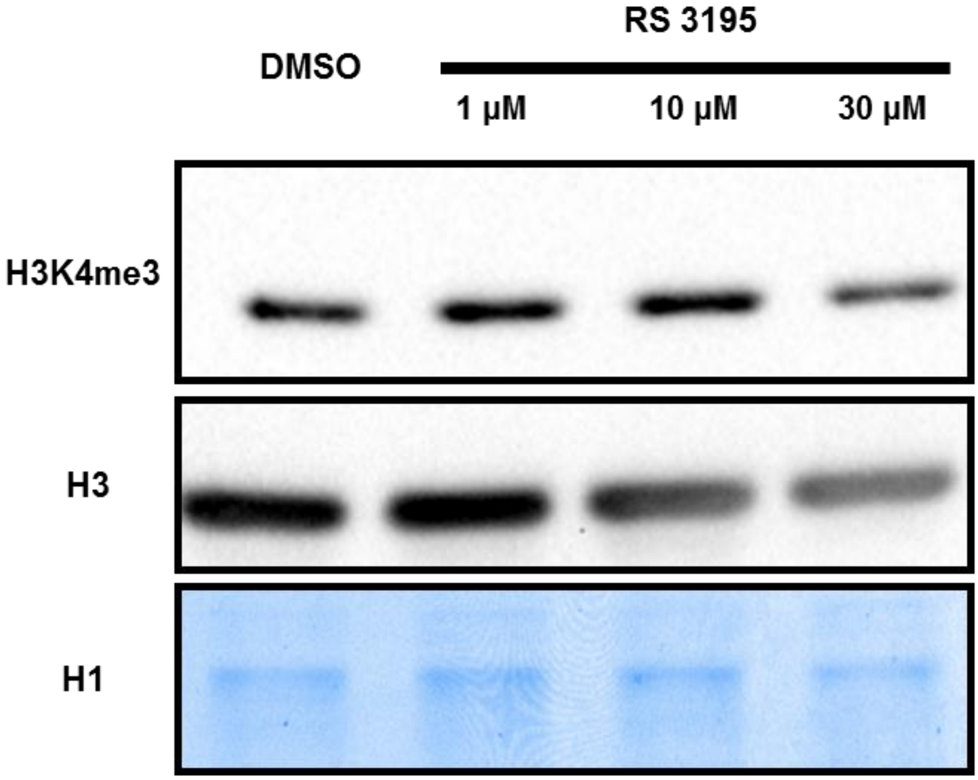

Figure S1. Western blotting analysis of H3K4me3 levels in MCF-7 cells incubated for 24 hours with three different concentrations of RS 3195. Shown image is representative from six independent experiments.

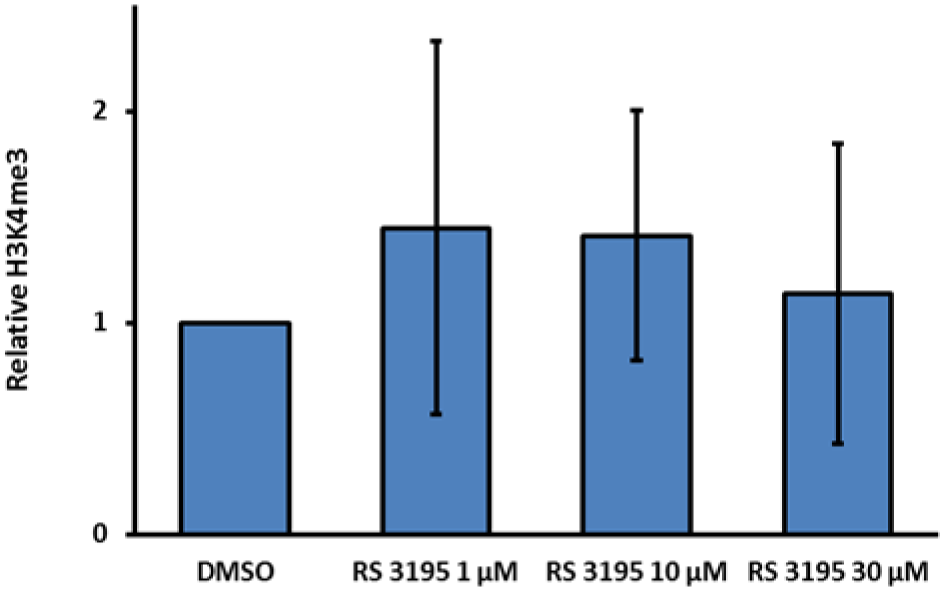

Figure S2. Quantification of six independent western blotting experiments, showing a slight increase in H3K4m3 levels upon RS3195 treatment. Data are normalized to DMSO treated control and represented as the mean ± SD of relative H3K4me3 levels.

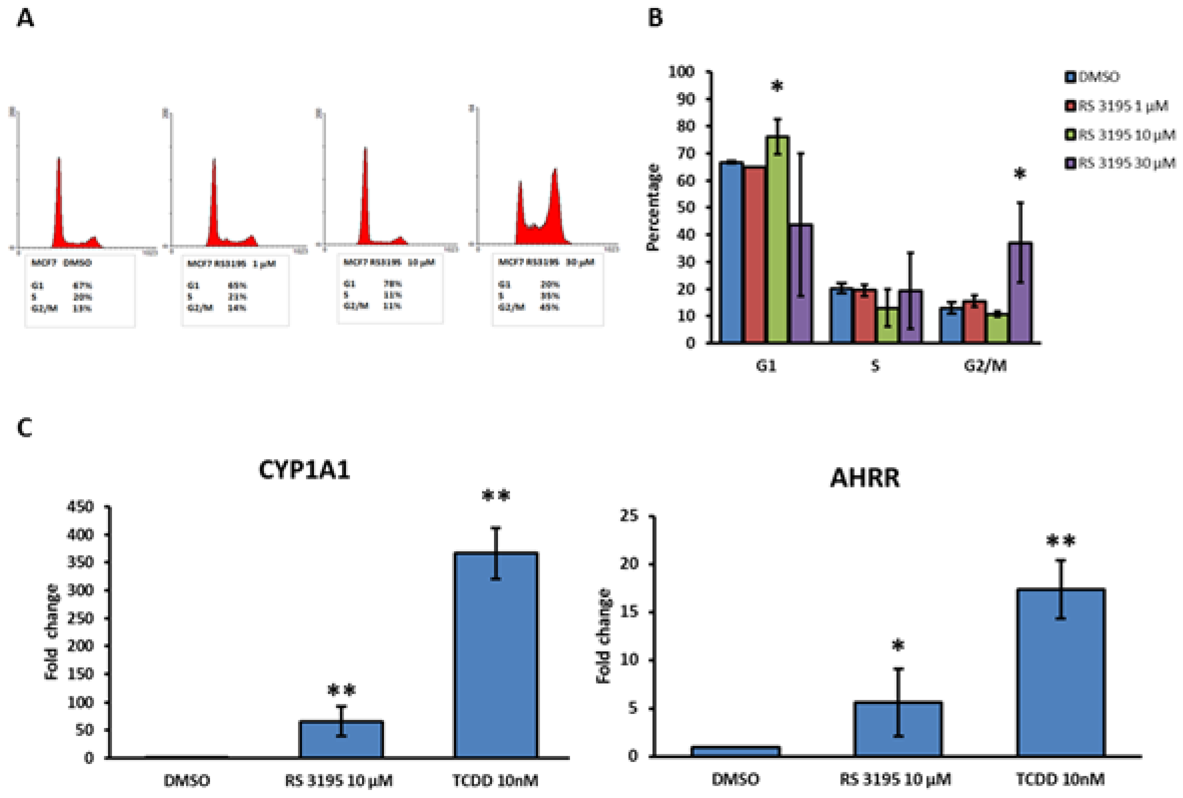

Figure S3. RS3195 is a KDM5 enzymes inhibitor that induces a strong G2/M arrest in MCF-7 cells. (A) Flow-cytometry analysis of cell-cycle distribution of MCF-7 cells treated with three different concentrations of RS 3195. The image is representative of three independent experiments. (B) Quantification of three independent experiments of flow cytometry analysis, indicating a strong increase in G2/M cells upon 30 µM RS3195 treatment. Data are represented as the mean ± SD of the corresponding percentage of cells in different cell cycle phases. (C) Transcript levels analysis by Real Time PCR of CYP1A1 and AHRR, in MCF-7 cells treated with 10 µM RS3195, indicating that this compound activates Aryl-hydrocarbon response. TCDD is used as control of the activation of AhR response as it is considered the best-known elicitor of the pathway. Data are represented as the mean ± SD of mRNA levels fold change from four independent experiments with respect to DMSO control. Statistical significance was assessed according to two-tailed paired Student’s t test. * $p<0.05$; ** $p<0.001$.

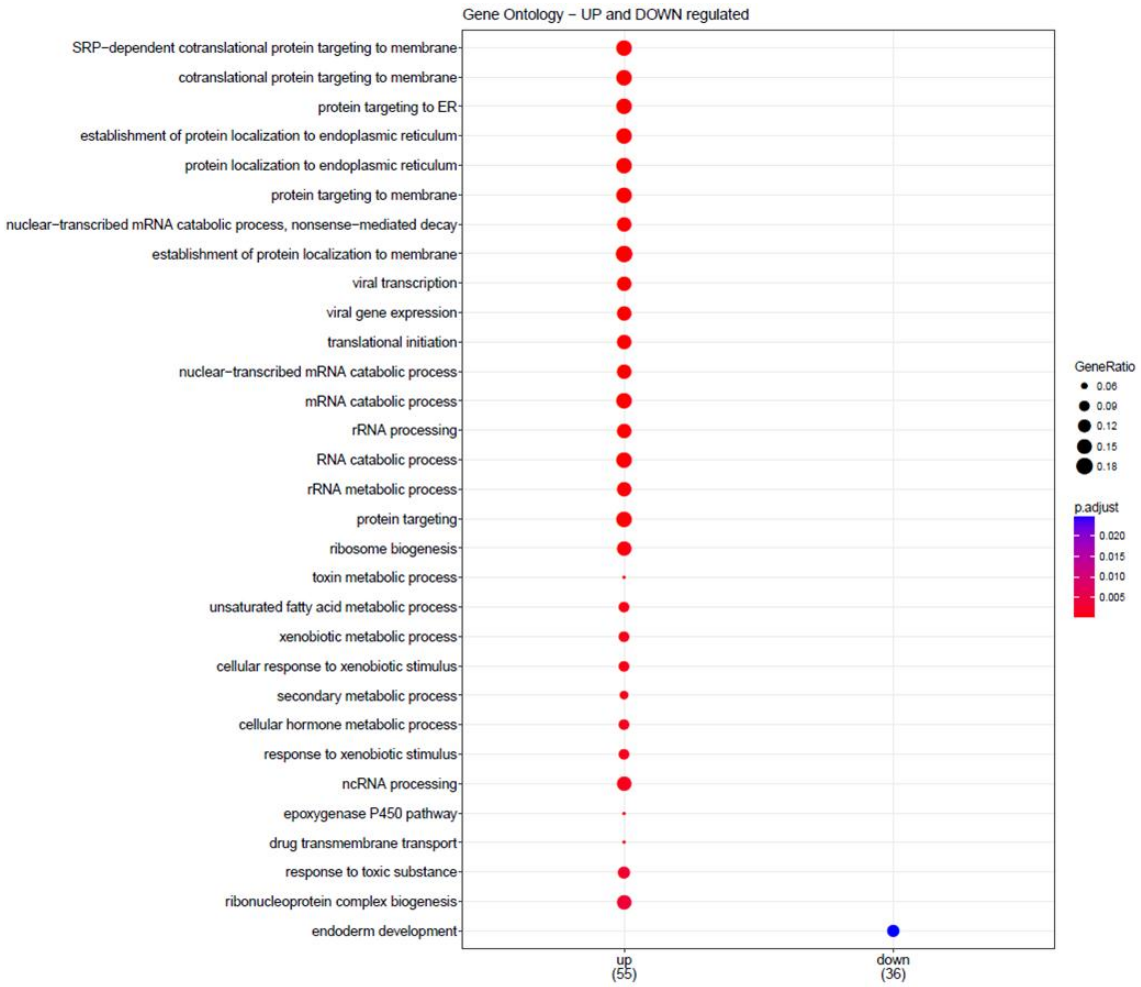

Figure S4. Dot plot representation of the results of Gene Ontology (Biological Process) analysis of RNA sequencing data from treated and untreated MCF7 cell samples, showing the categories of biological processes most enriched in genes upregulated or downregulated in treated MCF7 cell samples.

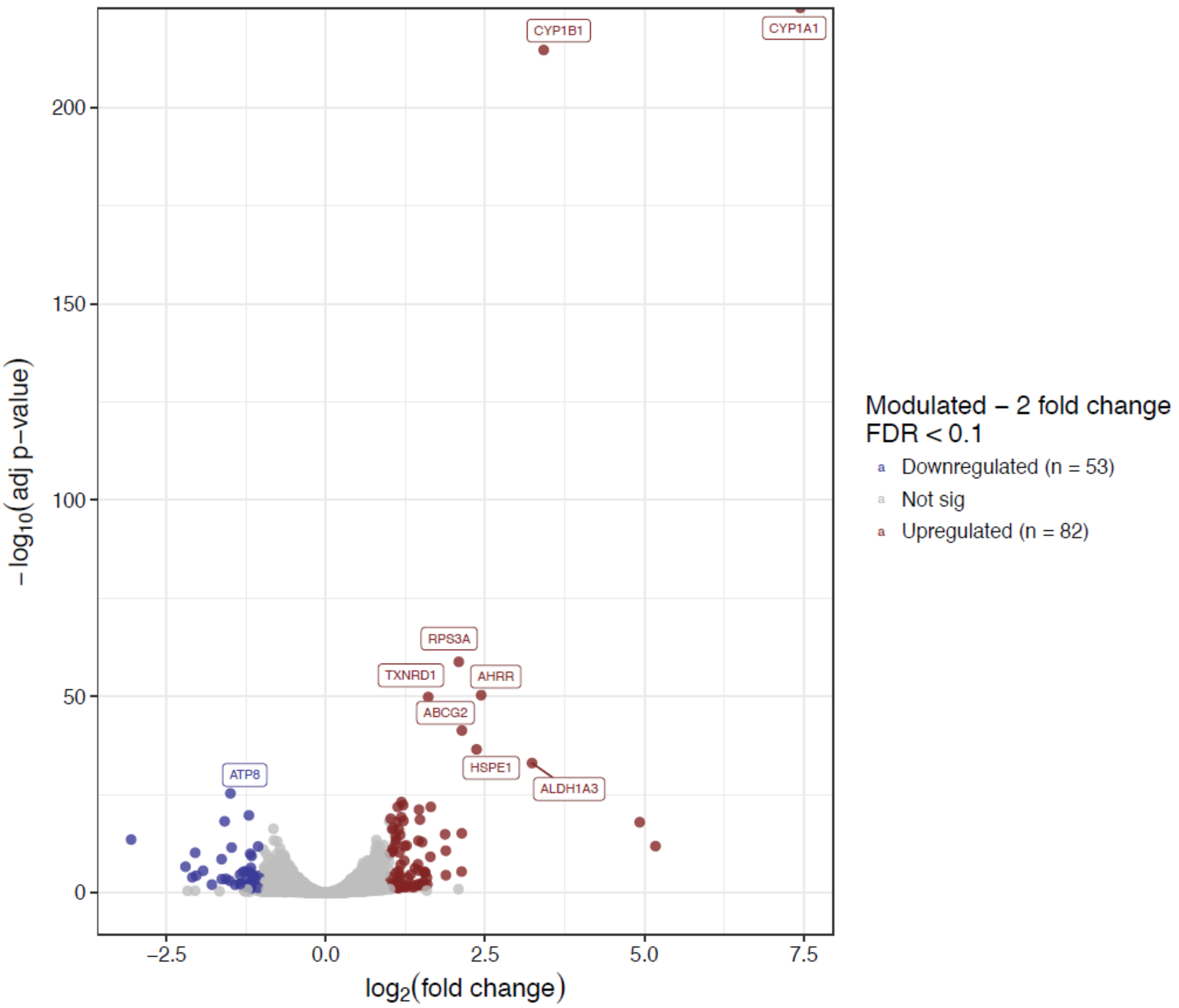

Figure S5. Volcano plot of log2 fold-change (x-axis) versus −log10 q-value (y-axis, representing the probability that the gene is differentially expressed) of the results of RNA sequencing analysis of treated and untreated MCF7 cell samples. 135 genes were significantly downregulated (blue dots) or upregulated (red dots), in grey are reported not significant modulated genes.

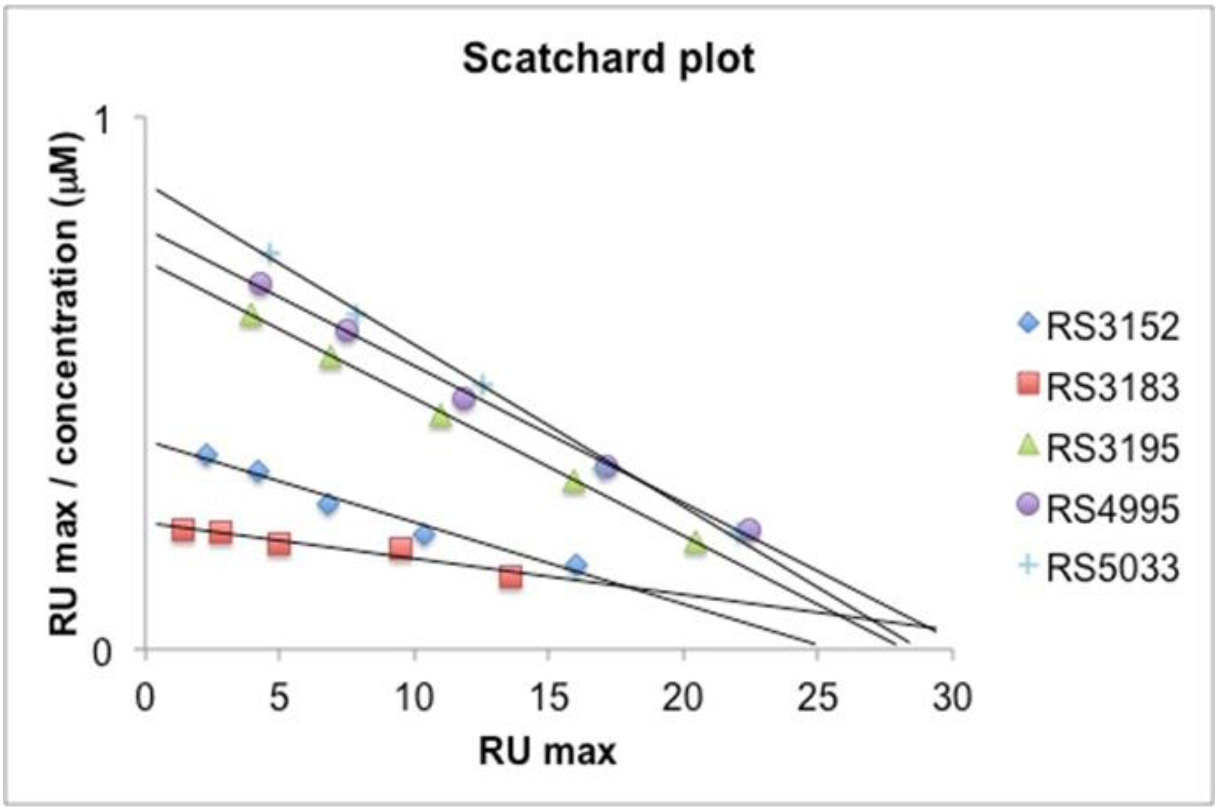

Figure S6. Scatchard plot of RS3152, RS3183, RS3195, RS4995, RS5033.

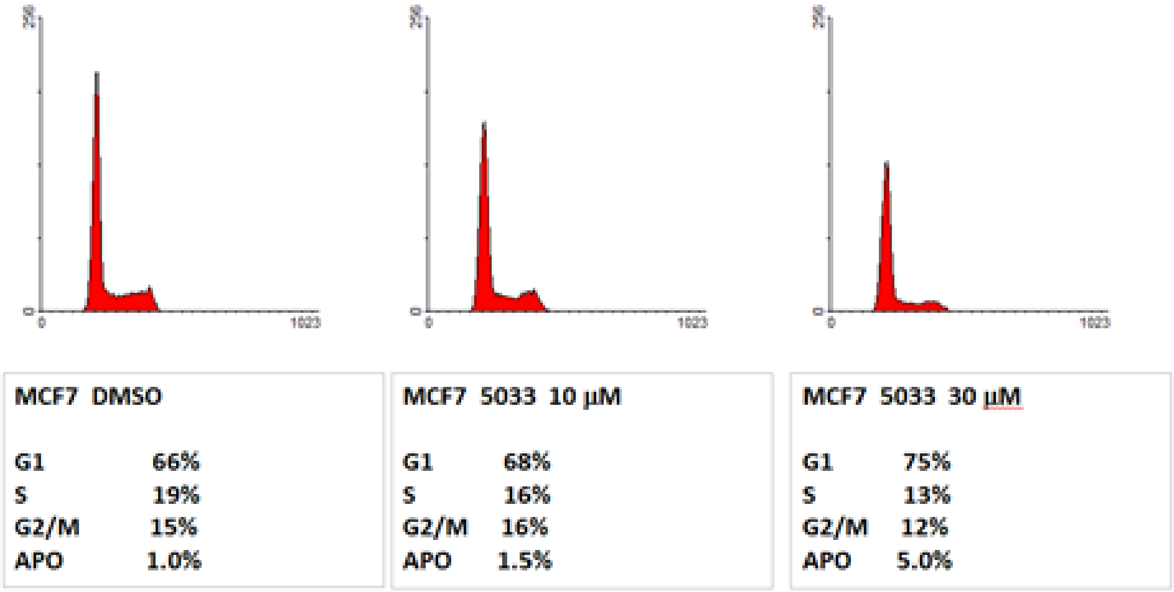

Figure S7. Flow-cytometry analysis of cell-cycle distribution of MCF-7 cells treated with two different concentrations of RS 5033. Shown image is representative from three independent experiments.

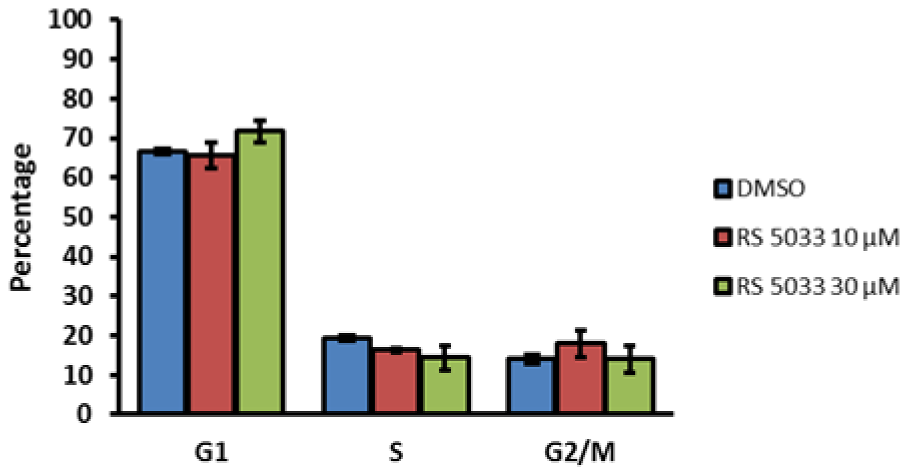

Figure S8. Quantification of three independent experiments of flow cytometry analysis, showing no significant perturbation of cell cycle dynamics by RS5033 treatment. Data are represented as the mean ± SD of the corresponding percentage of cells in a particular cell cycle phase.

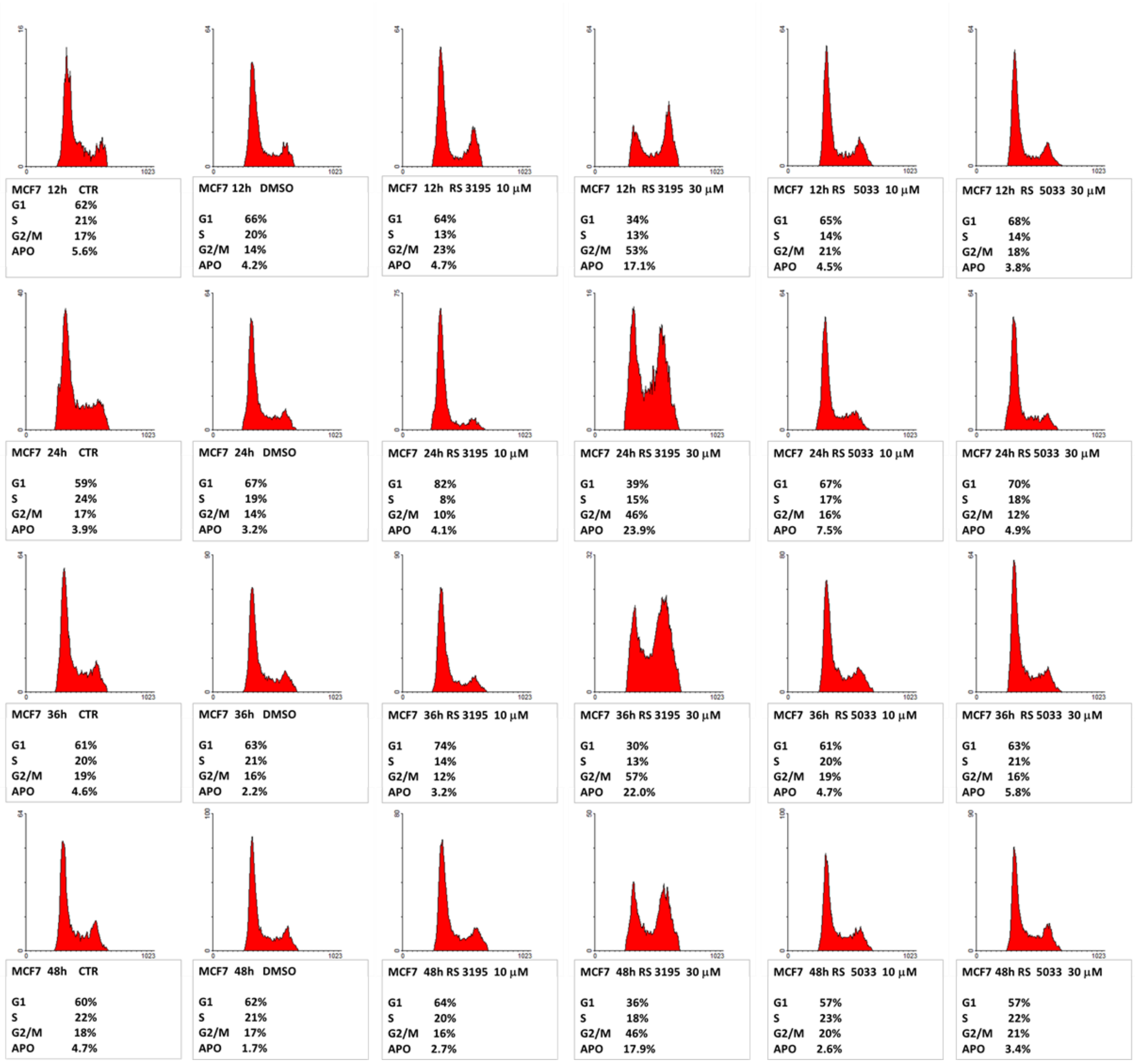

Figure S9. Comparative cytofluorimetric analysis of SubG1 population in MCF7 cells treated with RS 3195 and RS 5033. Cells treated with the indicated concentrations of RS 3195 and RS 5033 for the indicated times were stained with propidium and analyzed at cytofluorimeter as explained in Materials and Methods. The diagram reports the estimates of the % fraction of the cells in the various cell cycle phases and the % fraction of subG1 cells (Apo) which is suggestive (although not diagnostic) of induced apoptosis.

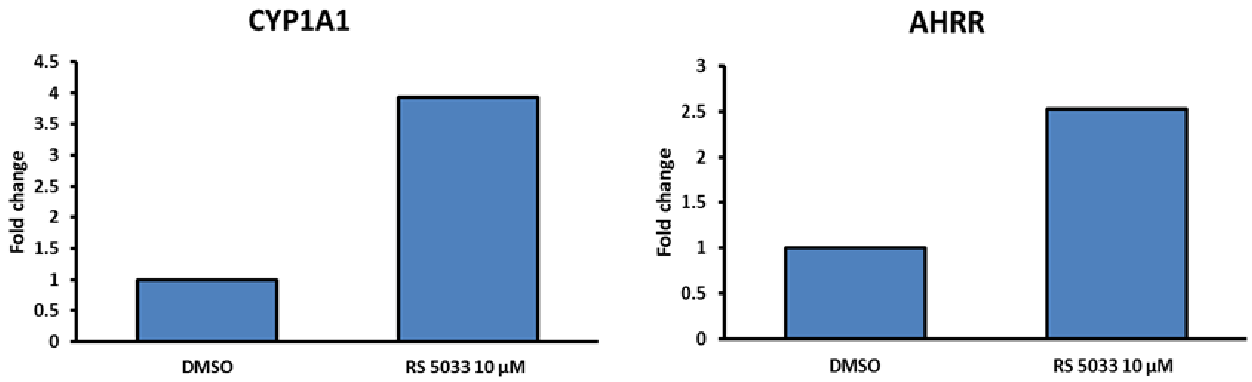

Figure S10. Transcript levels analysis by Real Time PCR of CYP1A1 and AHRR genes in MCF-7 cells treated with 10 μM RS5033, showing a lower increase of these genes compared to RS3195.

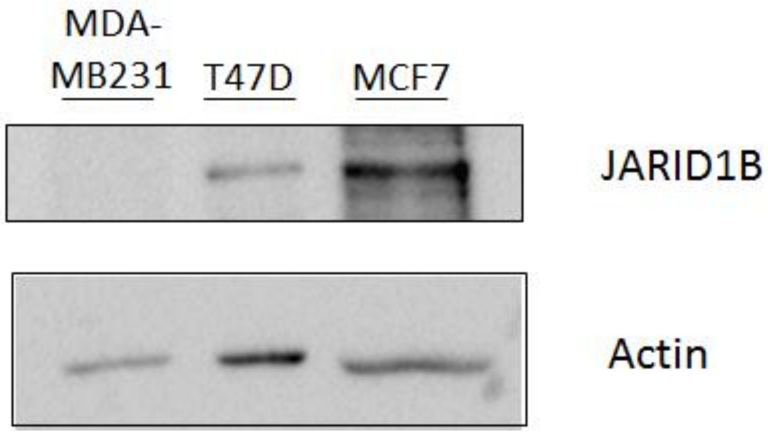

Figure S11. Western Blot showing the relative expression of Jarid1B in MCF-7, T47D and MBA-MB231 cell lines, respectively. Actin is reported as endogenous control.

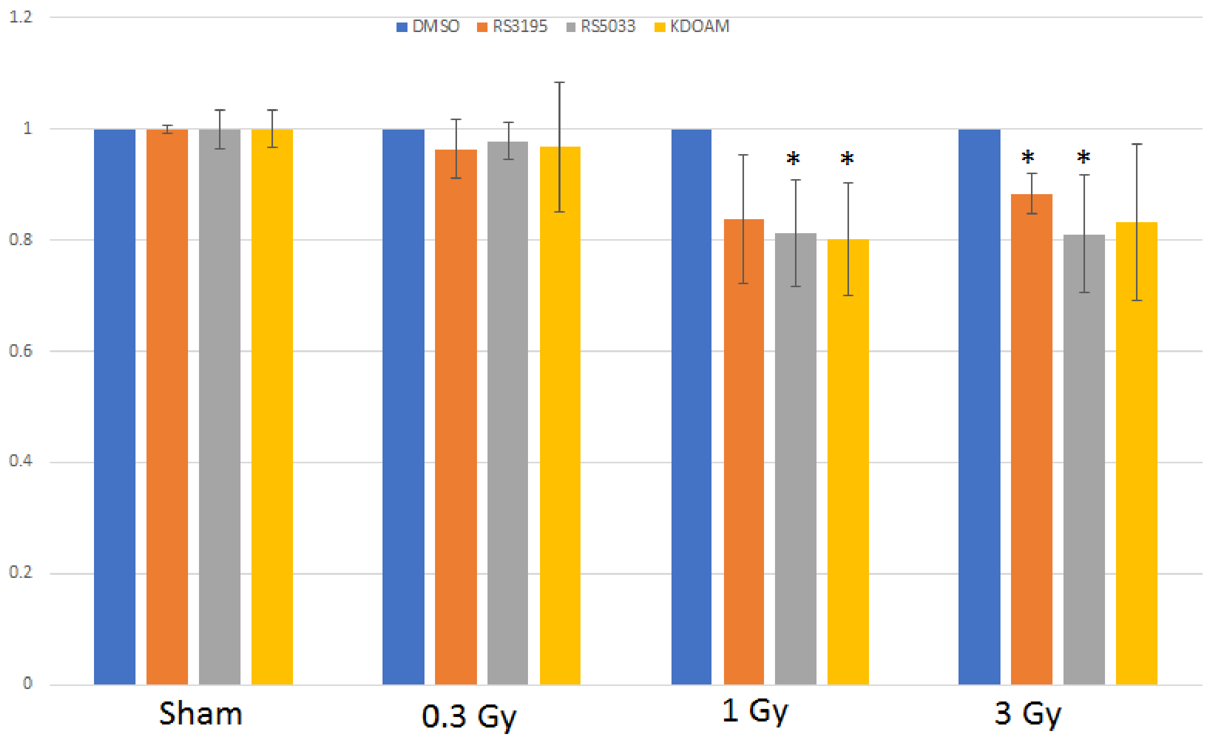

Figure S12. Viability assay on T47D breast cancer cells irradiated and treated with DMSO only (0.1%) (blue), 3 µM RS3195 (orange), 3 µM RS5033 (grey) or 0.3 µM KDOAM-25 (yellow). The histogram reports the mean of relative absorbance measured at 450 nm ± SD from three independent experiments. In order to appreciate the effect of the inhibitors on cell viability in each dose of radiation compared to DMSO control, the absorbance of each dose of radiation (sham, 0.3, 1 and 3 Gy) was normalized to the corresponding DMSO control and then, for each concentration of inhibitors, these values were normalized to the corresponding sham control of alive cells and represent the average. * = Pvalue <0.05 according to Student T-Test

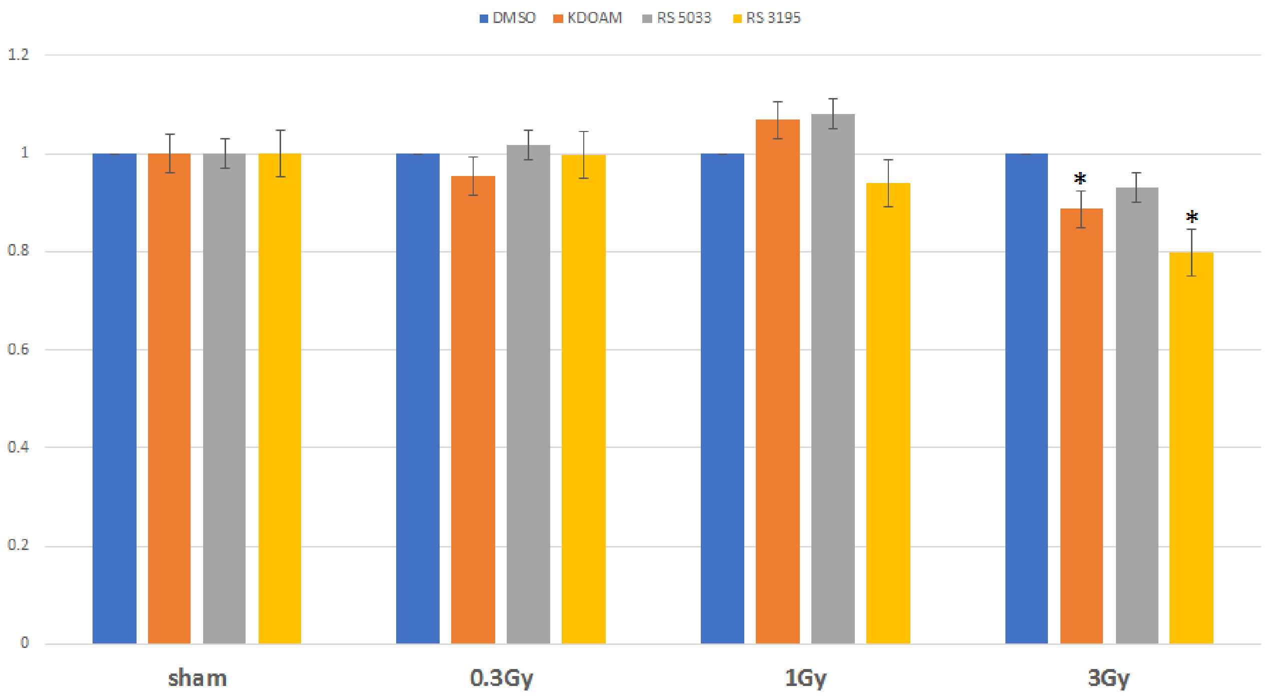

Figure S13. Viability assay on MDA-MB231 breast cancer cells irradiated and treated with DMSO only (0.1%) (blue), 3 μM RS 3195 (yellow), 3 μM RS 5033 (grey) or 0.3 μM KDOAM-25 (orange). The histogram reports the mean of relative absorbance measured at 450 nm ± SD from three independent experiments. In order to appreciate the effect of the inhibitors on cell viability in each dose of radiation compared to DMSO control, the absorbance of each dose of radiation (sham, 0.3, 1 and 3 Gy) was normalized to the corresponding DMSO control and then, for each concentration of inhibitors, these values were normalized to the corresponding sham control of alive cells and represent the average. * = Pvalue <0.05 according to Student T-Test

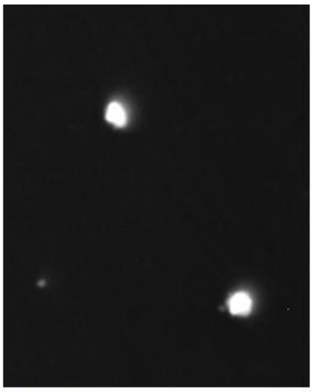

DMSO sham

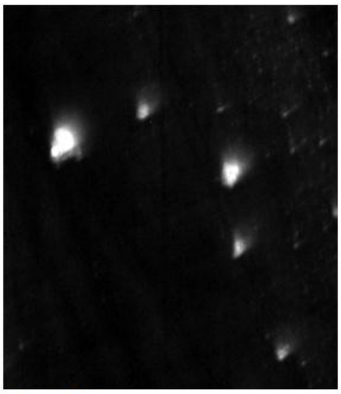

RS3195 sham

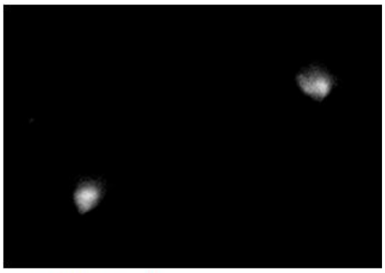

RS5033 sham

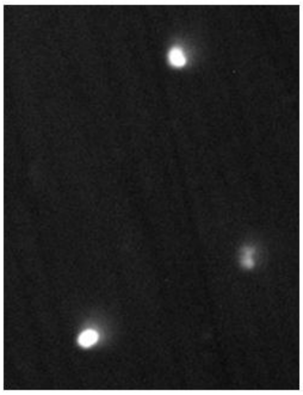

KDOAM25 sham

Figure S14. Selected COMET assay images from samples sham irradiated.

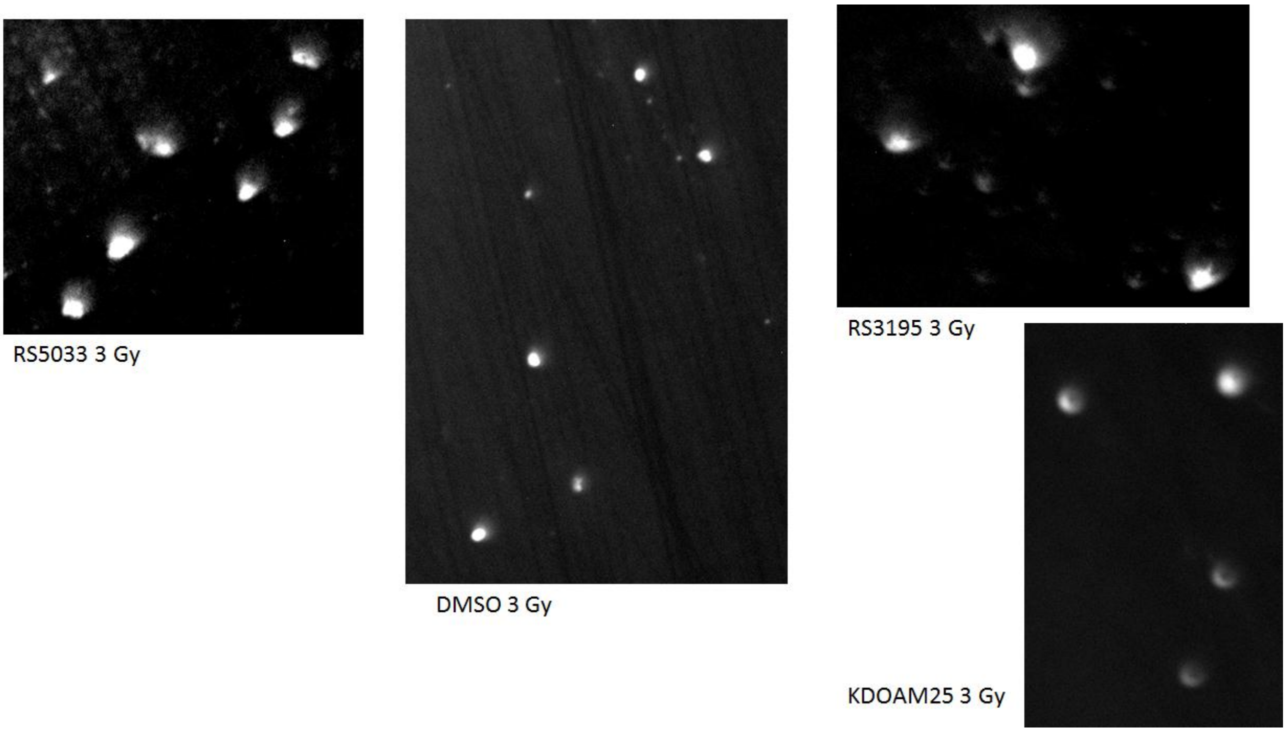

Figure S15. Selected COMET assay images from samples collected 1h after 3 Gy irradiation.

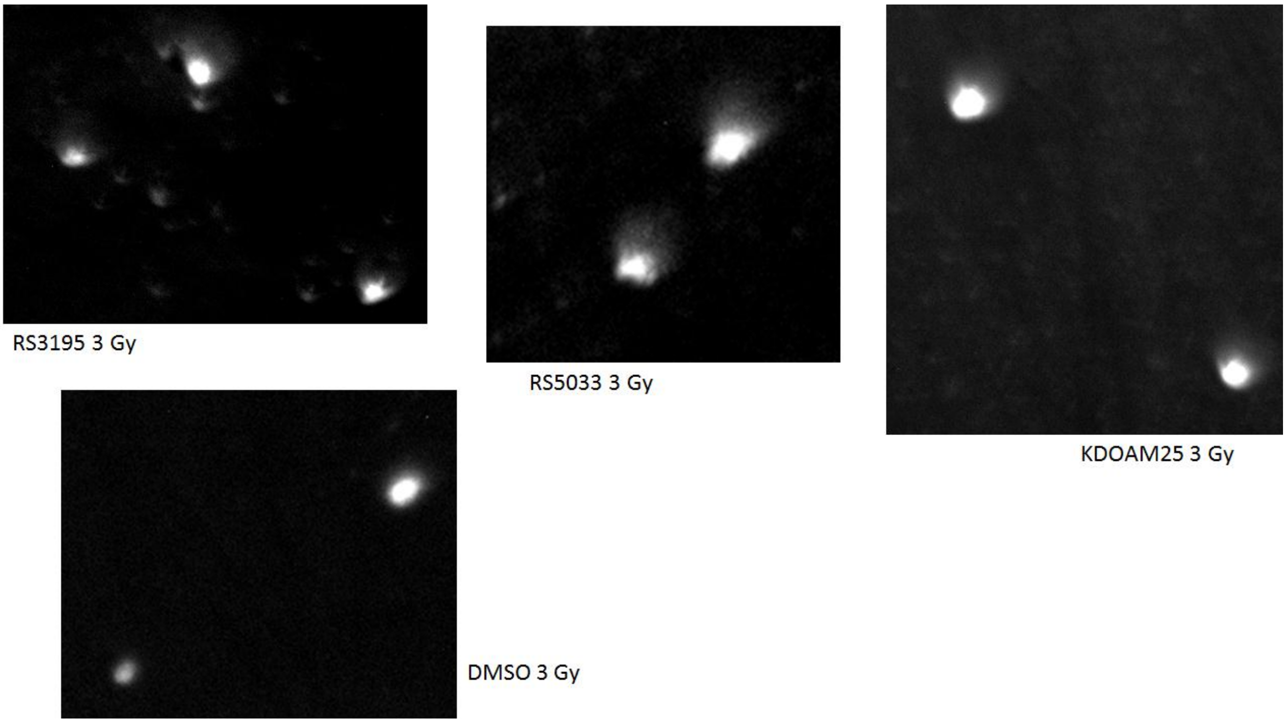

Figure S16. Selected COMET assay images from samples collected 1h after 3 Gy irradiation. Images are enlarged to better show tails.
